# Study on the coupling characteristics of different numbers of cavitation bubbles and particle groups in the free domain

**DOI:** 10.1016/j.ultsonch.2026.107838

**Published:** 2026-04-01

**Authors:** Shiqi Yang, Wei Han, Rennian Li, Xiaobo Shen

**Affiliations:** a School of Energy and Power Engineering, Lanzhou University of Technology, Lanzhou 730050, China; b School of Green Energy and Storage, Lanzhou University of Technology, Lanzhou 730050, China; c Key Laboratory of Advanced Pumps Valves and Fluid Control System of the Ministry of Education, Lanzhou University of Technology, Lanzhou 730050, China; d Key Laboratory of Fluid Machinery and Systems, Lanzhou 730050, China

**Keywords:** Free field, Cavitation bubble, Particle groups

## Abstract

In a free field multiphase environment, the coupling between cavitation bubble dynamics and particle groups can markedly reshape local flow structures and thereby influence particle impact risks. Within a unified dimensionless framework, this study investigates three representative systems—single cavitation bubble, double cavitation bubble, and three cavitation bubble—by combining experimental observations, theoretical analysis, and three-dimensional numerical simulations to systematically characterize the evolution of particle-group velocity magnitudes and directions. The results show that, for a single cavitation bubble, the maximum particle velocity decays significantly with increasing dimensionless distance, and the velocity direction points outward from the bubble center during expansion but reverses toward the center during collapse. For a double cavitation bubble, the particle response in the superposition region differs distinctly from that in the outer-side regions: when particles are located near the centerline connecting the bubble centers, the collapse-induced microjet can trigger peak acceleration events, with representative particle velocities reaching the order of 60–70 m/s, far exceeding those dominated by radiation pressure; meanwhile, the particle velocity direction in the superposition region can become approximately perpendicular to the centerline. For a three cavitation bubble, particle-group responses are highly sensitive to the configuration and the relative inter-bubble spacing parameters: symmetric configurations tend to exhibit more collective responses, whereas asymmetric or non-collinear configurations enhance the spatial expansion and dispersed contributions of the superposition region. Group-level statistics further indicate that multi-bubble systems can substantially strengthen y-direction momentum exchange, and a set of group metrics reveals systematic changes in contribution patterns and the dominant affected range. These findings clarify the region-dependent dominance of radiation pressure and microjet impact in cavitation bubble–particle groups coupling, providing reproducible quantitative evidence for particle impact assessment in multiphase systems.

## 1 Introduction

Cavitation has attracted extensive attention in recent years and is widely encountered in many fields, including chemical engineering [1], [2], biomedical applications [3], [4], and ocean engineering [5], [6]. When solid particles are present in the medium, cavitation can couple with particles, further increasing flow complexity and significantly raising the difficulty of related studies. In particular, in fluid machinery [7], [8], [9], cavitation-accelerated sediment erosion is recognized as one of the major challenges faced by hydraulic turbines [10]. According to an investigation [11], more than one third of hydraulic turbines in China have suffered damage induced by the synergistic action of cavitation and particles. Brekke et al. [12] reported that severe erosion occurs when cavitation and sediment erosion coexist. The fundamental reason is that, in particle-laden flows, the synergy between cavitation and particles can intensify cavitation erosion, thereby exacerbating fatigue and damage of wetted walls. Zhang et al. [13], [14], through cavitation erosion and wear experiments on hydrofoils in sediment-laden water, found that the damage caused by the synergistic effect is markedly greater than that caused by either mechanism alone. They further revealed that this intensification is closely related to the pronounced acceleration of sediment particles driven by pressure waves and microjets. Consequently, many studies have focused on macroscopic interactions between cavitation and particles [15], [16], [17], [18], such as correlations between damage and particle properties, showing that erosion inside hydraulic turbines strongly depends on sediment characteristics (e.g., size, hardness, concentration, and surface morphology), internal flow conditions, and material properties [10]. Moreover, cavitation-induced unsteady fluctuations and two-phase effects are not limited to large-scale fluid machinery; they are also prevalent in engineering scenarios such as microscale pumping and multiphase transport. For example, investigations on ferrofluid micropumps—including flow-rate characteristics and velocity pulsation behavior, pumping mechanisms in confined geometries, and experimentally validated models—have demonstrated that two-phase structures can substantially alter unsteady responses and performance boundaries [19], [20], [21]. In addition, the cavitation bubble generation mechanism and the influence of key parameters on performance further highlight the universality and complexity of cavitation bubble–coupling problems in engineering systems [22].

To elucidate the synergistic mechanism, macroscopic studies alone are far from sufficient; microscopic investigations are essential for a deeper understanding of the interactions between cavitation and particles. Starting from the canonical problem of a single cavitation bubble interacting with a single particle, extensive efforts have been devoted to clarifying the underlying mechanisms, with particular emphasis on particle size and concentration [23], [24], particle shape [25], [26], the relative size between the particle and the cavitation bubble [27], and the distance between the cavitation bubble and the particle [28]. Li [29] proposed a microscopic model for the synergistic effect between cavitation and solid particles, suggesting that particles can be accelerated to extremely high velocities under the impact of a jet core. Lv [30] experimentally demonstrated that, at relatively large separations, particles may be repelled from or attracted toward a cavitation bubble during its expansion and collapse, a phenomenon closely associated with radiation pressure induced by the cavitation bubble; at smaller separations, particles may be accelerated by microjet impact. Poulain et al. [31] suspended spherical particles using a thread and also observed particle motion away from or toward the cavitation bubble; they further summarized the relationship between particle velocity and particle density. Wu et al. [32] reported that cavitation bubble growth can accelerate freely settling particles. Building on these studies, Shen et al. [33] improved the experimental conditions to investigate the interaction between a single cavitation bubble and completely unconstrained, fully suspended particles in an unbounded domain, and derived an analytical expression for particle acceleration induced by a single cavitation bubble in free field. These studies provide a microscopic explanation for the macroscopic observation that particle-laden flows exacerbate surface damage in low-pressure cavitating regions: the cavitation bubble accelerates surrounding particles, resulting in higher wear rates on surfaces.

In practice, cavitation bubbles rarely exist in isolation; they more commonly appear as cavitation clouds or multiple cavitation bubbles acting simultaneously. As the number of cavitation bubbles increases, more complex dynamic behaviors emerge due to inter-bubble interactions. Fong et al. [34] investigated the interaction between a double cavitation bubble and classified the interaction patterns based on their observations. Chew et al. [35] studied the interaction between two cavitation bubbles of different sizes in a free field, and identified four typical behaviors: mutual jetting, unidirectional jetting, coalescence, and a ejection effect. Han et al. [36] further examined the interaction of two laser-induced bubbles and showed that jet intensity and direction can be controlled by adjusting the relative bubble positions, the time delay between bubble generation, and laser pulse energy. Numerical simulations have also been used to study the coalescence of two cavitation bubbles into a single merged cavitation bubble, revealing that the liquid-film thickness between the two cavitation bubbles determines the coalescence criterion [37]. These studies have clarified that the inter-bubble distance (i.e., liquid-film thickness), phase difference, and relative bubble size are key factors governing the interaction mode and intensity in a double cavitation bubble system. Han et al. [38], based on incompressible potential-flow theory and the boundary integral method, investigated the dynamics of two cavitation bubbles with different sizes and, by introducing a vortex-ring model, simulated the subsequent evolution of two toroidal bubbles after a jet penetrates both cavitation bubbles. Importantly, after collapse in a free-field double cavitation bubble system, the ensuing flow features differ from those of a single cavitation bubble, which may further modify the intensity of cavitation bubble–particle coupling. Yang et al. [39] conducted experimental studies on single-particle dynamics under a double cavitation bubble and a three cavitation bubble, and found that increasing the number of cavitation bubbles leads to particle dynamics that differ from those under a single cavitation bubble. Xie et al. [40] performed theoretical investigations on the dynamics of a spatial three cavitation bubble system and showed that interactions among symmetrically positioned cavitation bubbles are jointly governed by the dimensionless distance and the arrangement geometry. Within a dimensionless framework, they also analyzed the effects of spatial configuration and flow compressibility, particularly noting that, for symmetric three-bubble arrangements, compressibility can dominate the behavior of the central cavitation bubble. Yang et al. [41], [42] further reported that, in a three cavitation bubble system, the relative magnitude of inter-bubble spacing between neighboring cavitation bubbles is a key factor influencing jet intensity.

Although significant progress has been achieved in studies of double cavitation bubble and three cavitation bubble systems, and these results provide important foundations for theoretical calculation and analysis, research on their coupling with particles remains limited—especially for cavitation bubble interactions with particle groups, which have rarely been reported. For a single cavitation bubble in free field, the flow field remains symmetric, and particle dynamics depend primarily on distance-related parameters. However, increasing the number of cavitation bubbles breaks the original symmetry of the single cavitation bubble flow field. For example, in a double cavitation bubble flow field, there exists an overlap region affected by both cavitation bubbles. Although Yang et al. [39] conducted a preliminary investigation on a double cavitation bubble interacting with a single particle, particles located in the intermediate region between the two cavitation bubbles were not considered. Moreover, Chew et al. [35] showed that a double cavitation bubble system can exhibit multiple interaction scenarios, and the corresponding particle responses remain unclear. The three cavitation bubble problem is even more complex, as different spatial distributions and positional parameters can substantially influence particle dynamics. Furthermore, existing studies suggest that particles are not merely passive responders; their presence may reshape cavitation-induced jet patterns and thresholds, indicating stronger nonlinear feedback [43]. Meanwhile, theoretical frameworks such as Kelvin impulse, used to characterize cavitation bubble migration and motion near solid particles, also reflect both the complexity and the predictability limits of cavitation bubble dynamics under bubble–particle coupling [44].

Despite substantial advances in cavitation bubble dynamics, existing studies have predominantly focused on the evolution characteristics of single cavitation bubble, double cavitation bubble, and three cavitation bubble systems, while particle-coupling effects have received comparatively less attention. Most available work has concentrated on single-particle responses driven by a single cavitation bubble, whereas particle responses under coupled double cavitation bubble and three cavitation bubble conditions still lack systematic investigation. Moreover, in practical multiphase environments, particles often exist in the form of particle groups, where inter-particle interactions and collective synergistic responses are more complex; related studies remain at an early stage. In this context, revealing the coupling dynamics between cavitation bubbles and particle groups in free field is of great significance for improving multiphase-flow theory and guiding engineering applications. During cavitation bubble collapse, radiation forces and microjets can strongly affect particle migration, clustering, and detachment. In addition, the direct influence of cavitation-related shock waves and acoustic-streaming shear in ultrasonic fields on dispersed-phase structures further supports the universality of cavitation effects and distance effects in multiphase systems [45], [46]. Conversely, the feedback of the dispersed phase on cavitation-induced flow structures further strengthens the nonlinearity and spatiotemporal coupling of such systems [43].

Within multi-cavitation-bubble systems, the present study places particular emphasis on the three cavitation bubble configuration. Although a double cavitation bubble system can describe pairwise coupling, its geometric symmetry and limited degrees of freedom make it difficult to represent the non-collinear distributions and unequal spacing commonly observed in cavitation bubble clouds. A three cavitation bubble system constitutes the smallest nontrivial configuration that introduces geometric asymmetry and unequal spacing and can trigger genuine many-body coupling effects, thereby serving as a bridge between double cavitation bubble studies and more complex cavitation-bubble-cloud problems. Accordingly, representative configurations of single cavitation bubble, double cavitation bubble, and three cavitation bubble systems (including collinear and triangular arrangements) are considered to investigate cavitation bubble–particle groups coupling dynamics in free field. Experimental observations are used to characterize the dynamic responses of particle groups driven by systems ranging from single cavitation bubble to three cavitation bubble in free field, while theoretical analysis provides preliminary validation and mechanistic interpretation. In addition, three-dimensional numerical simulations are conducted to track and compare the forces and trajectories of representative particles from a multiscale perspective, thereby elucidating the spatiotemporal evolution of cavitation bubble–particle groups systems in complex flow fields. The results provide a basis for cross-scale understanding of cavitation bubble–particle groups coupling dynamics and offer references for engineering applications such as ultrasonic cleaning, cavitation erosion mitigation, and biofluid processing.

## 2 Experimental process

### 2.1 Introduction to the experimental setup

**Fig. 1:**
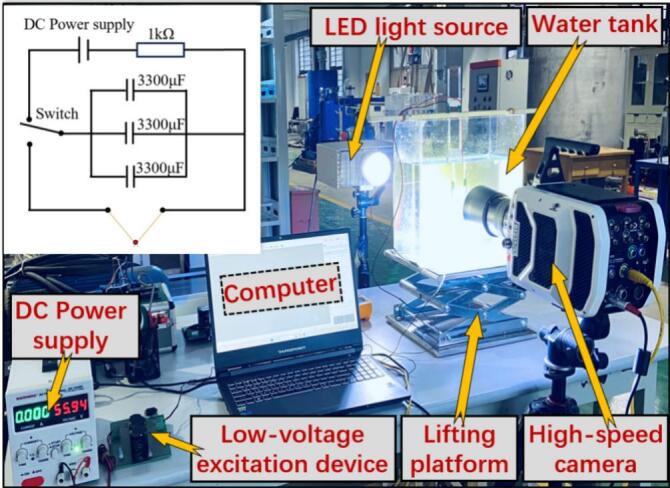
Physical diagram of the experimental setup.

To investigate the dynamics of cavitation bubbles interacting with particle groups in a free field, a series of experiments on cavitation bubble–particle group interactions were conducted in this study. Photographs of the major components of the experimental setup are provided in Fig. 1. The systems and key performance parameters are described as follows. The imaging system consisted of a Phantom v1210 high-speed camera coupled with a Canon EF 100 mm f/2.8 L macro lens. The imaging parameters were set to a resolution of 512 × 512 pixels and an acquisition rate of 37,514 fps, with an exposure time of 26.7 μs per frame. To enhance flow visualization quality, a 300 W LED lamp was used as the illumination source to provide uniform and high-intensity lighting, ensuring that the imaging system could clearly resolve the motion of the cavitation bubble and particles. Considering the requirement for adjustable cavitation bubble position parameters, cavitation bubbles were generated using a spark-discharge method. The discharge system employed a parallel electrolytic capacitor bank with a total capacitance of 9900 μF (rated voltage 100 V per capacitor). Charging and discharging were controlled via a single-pole double-throw switch, and the system was powered by a 0–100 V adjustable DC power supply (maximum power 300 W). To minimize additional effects of electrode diameter on cavitation bubble behavior, a 0.2 mm copper wire, whose diameter was much smaller than the characteristic cavitation bubble size, was used as the discharge electrode.

**Fig. 2:**
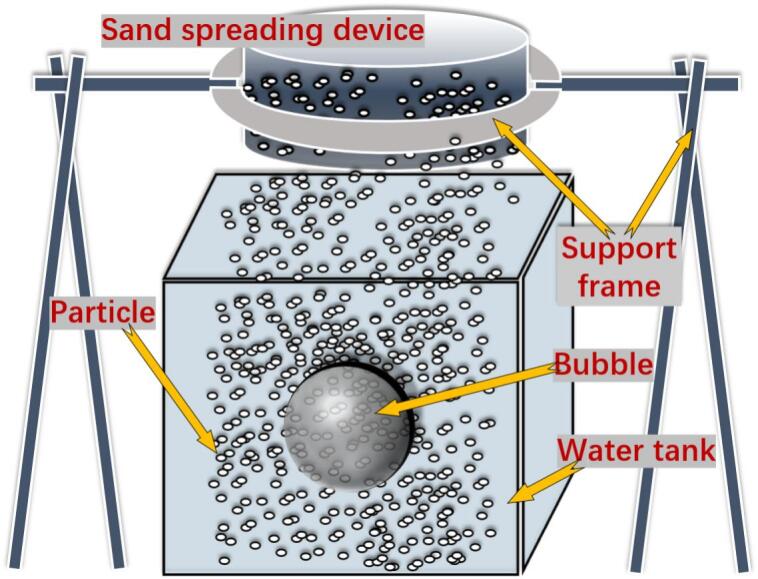
Schematic diagram of the device for the coupling effect between cavitation bubbles and particle groups.

In addition, the key component for realizing cavitation bubble–particle group coupling is the interaction apparatus shown in Fig. 2. It mainly consists of three parts: a water tank, a supporting frame, and a sand feeder. With the supporting frame, the sand feeder was fixed directly above the water tank. By continuously adding sand into the feeder, a randomly distributed sand field was formed in the tank. Then, by closing the switch of the discharge system, a cavitation bubble was generated within the sand field, thereby enabling experiments on cavitation bubble–particle group coupling. The particles used were silicon carbide, with a mesh size range of 26–40 mesh (0.43–0.71 mm).

### 2.2 Experimental parameters

To facilitate the description of distance parameters between cavitation bubbles and particles, the inter-particle and cavitation bubble distances were nondimensionalized prior to the study. Considering that the three cavitation bubble configuration inherently includes scenarios of single and double cavitation bubbles, two examples of three cavitation bubble systems were selected to illustrate the dimensionless parameters.

**Fig. 3:**
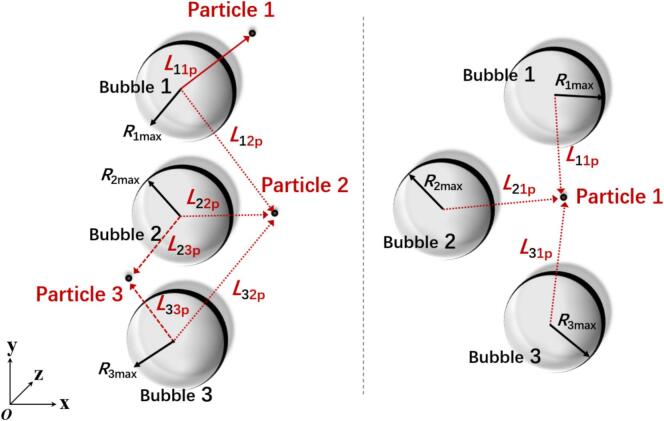
Coupling parameter diagram of cavitation bubbles and particle clusters.

As shown in Fig. 3(a), particle 1 is primarily influenced only by cavitation bubble 1. Particles that are affected solely by their adjacent cavitation bubbles are classified as the first type, and all particles in a single cavitation bubble system fall into this category. In Fig. 3, particle 3 is mainly affected by cavitation bubbles 2 and 3 and is therefore classified as the second type. In a double cavitation bubble system, the particle group is mainly composed of first- and second-type particles. Particle 2, influenced by cavitation bubbles 1, 2, and 3, is classified as the third type, which represents the dominant particle type in the three cavitation bubble system. In Fig. 3(b), corresponding to the triangular arrangement of three cavitation bubbles, particle 1 is affected by the combined influence of all three cavitation bubbles and thus belongs to the third type. The dimensionless distance between cavitation bubble i and particle j is denoted as *λ*_ijp,_ which is defined as follows:

(1) $$\lambda_{\mathrm{ijp}}=\frac{L_{\mathrm{ijp}}}{R_{\mathrm{imax}}}$$

Among them, *L*_ijp_ represents the actual size between the cavitation bubble i and the particle j, while *R*_imax_ represents the maximum radius of the cavitation bubble i. Additionally, for the cases of double cavitation bubbles and three cavitation bubbles, a dimensionless parameter *λ*_mn_, which characterizes the distance between the cavitation bubbles, needs to be introduced. Its specific expression is as follows:

(2) $$\lambda_{\mathrm{mn}}=\frac{L_{\mathrm{mn}}}{R_{(m)max}+R_{(n)max}}\left(m\neq n\right)$$

Among them, *L*_mn_ represents the actual size between cavitation bubble m and cavitation bubble n, while *R*_(m)max_ represents the maximum radius of cavitation bubble m. *R*_(n)max_ represents the maximum radius of cavitation bubble n, where m ≠ n.

### 2.3 Analysis of experimental device stability

To evaluate the repeatability and stability of the experimental setup, experiments were performed at six different charging voltages. For each voltage condition, five independent measurements were conducted. The maximum cavitation bubble radius in each experiment was measured using Image J, and the mean value, standard deviation, and relative uncertainty were then calculated. The corresponding results are summarized in Table 1. The analysis indicates that the excitation system exhibits good overall stability, with the relative uncertainty maintained within 3.24%–5.85%. Such low uncertainty confirms that the experimental setup and ambient conditions meet the requirements for repeatable tests, thereby providing a reliable experimental data basis for subsequent investigations of cavitation bubble–particle group coupling in a free field. The results are as follows:

**Table 1:** Uncertainty Analysis of the Experimental Excitation Device.

Voltage	45 V	50 V	55 V	60 V	65 V	70 V
*R*_ave_(mm)	4.953	5.240	6.543	7.170	7.879	8.544
Standard deviation	0.227	0.281	0.212	0.419	0.384	0.307
Relative uncertainty (%)	4.59	5.36	3.24	5.85	4.87	3.59

## 3 Theoretical analysis and experimental phenomena

### 3.1 Theoretical analysis

For the problem of a single cavitation bubble in a free domain, its effect is modeled as a point source with intensity *Q*(t) = 4π*R*_b_^2^ $\dot{R_{b}}$ [47], where *Q*(t) represents the rate of volume change. This point source is designated as Point Source 1. It is also observed that *Q*(t) corresponds to the rate of change of the cavitation bubble's volume, *Q*(t) = d*V*_b_**/**d*t*. The cavitation bubble is considered a pure steam bubble. To derive the time-dependent radius of the collapsing cavitation bubble, *R*_b_(t), the Keller-Miksis bubble dynamics equation [48] is applied.

(3) $$\left(1-\frac{{\dot{R}}_{1}}{c}\right)R_{1}{\ddot{R}}_{1}+\left(\frac{3}{2}-\frac{{\dot{R}}_{1}}{2c}\right){\dot{R}}_{1}^{2}=\left(1+\frac{{\dot{R}}_{1}}{c}\right)\frac{P_{R1}-p_{0}}{\rho }+\frac{R_{1}}{\rho c}\frac{d\left(P_{R1}-p_{0}\right)}{\mathrm{dt}}$$

The expression of *P*_R1_ is as follows:

(4) $$P_{R1}=\left(p_{0}-p_{\nu }+\frac{2\sigma }{R_{0}}\right){\left(\frac{R_{0}^{3}-bR_{0}^{3}}{R^{3}-bR_{0}^{3}}\right)}^{k}-\frac{2\sigma }{R}-\frac{4\mu \dot{R}}{R}+p_{\nu }$$

**Fig. 4:**
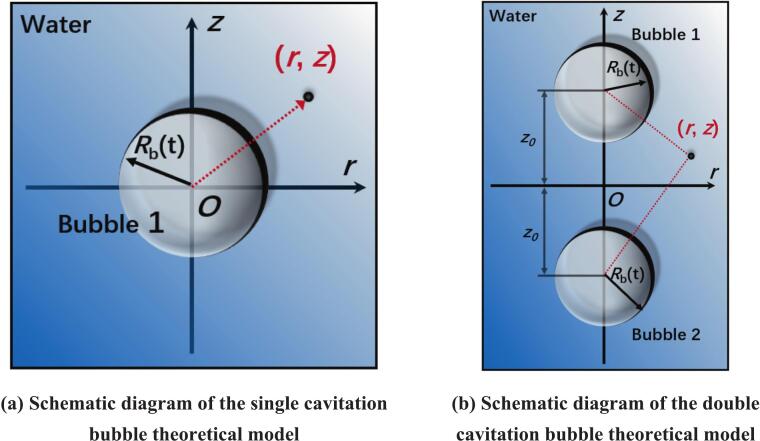
Flow field model of cavitation bubbles under different numbers.

In the cylindrical coordinate system, the schematic diagram of the single cavitation bubble theory model is shown in Fig. 4(a). The velocity potential generated by the single cavitation bubble 1 at any position (*r, z*) in the flow field is expressed as

(5) $$\Phi_{1}=-\frac{Q}{4\pi \|\left(r,z\right)-\left(r_{0},z_{0}\right)\|}=-\frac{4\pi R_{b}^{2}\dot{R_{b}}}{4\pi {\left(r^{2}+z^{2}\right)}^{\frac{1}{2}}}$$

The velocity potential at any position (*r, z*) in the flow field is solely due to the point source 1. Therefore, there is

(6) $$\Phi =\Phi_{1}$$

The velocity ***U***=(*U*_r_,*U*_z_) is the vector sum of the spatial gradients of each velocity potential, and its expression is

(7) $$U_{r}=\frac{d\Phi_{1}}{\mathrm{dr}}$$

(8) $$U_{z}=\frac{d\Phi_{1}}{\mathrm{dz}}$$

Finally, the result obtained is:

(9) $$U_{r}=R_{b}^{2}{\dot{R}}_{b}\frac{r}{{\left(r^{2}+z^{2}\right)}^{\frac{3}{2}}}$$

(10) $$U_{z}=R_{b}^{2}{\dot{R}}_{b}\frac{z}{{\left(r^{2}+z^{2}\right)}^{\frac{3}{2}}}$$

For the problem of double cavitation bubbles, their effect can be equivalently regarded as two point sources. As shown in Fig. 4(b), the intensity of the point source *Q_i_*_(_t) = 4π*R*_b_^2^ $\dot{R_{b}}$. Correspondingly, for the velocity potential, it becomes the superposition of the intensities of the two point sources. The velocity potential at any position (*r, z*) in the flow field is the result of the superposition of point source 1 and point source 2. Therefore, there is

(11) $$\Phi =\Phi_{1}+\Phi_{2}$$

(12) $$\Phi_{1}=-\frac{Q}{4\pi \|\left(r,z\right)-\left(r_{0},z_{0}\right)\|}=-\frac{4\pi R_{b}^{2}\dot{R_{b}}}{4\pi ({\left({r-r_{0})}^{2}+{\left(z_{0}-z\right)}^{2}\right)}^{\frac{1}{2}}}$$

(13) $$\Phi_{2}=-\frac{Q}{4\pi \|\left(r,z\right)-\left(r_{0},{-z}_{0}\right)\|}=-\frac{4\pi R_{b}^{2}\dot{R_{b}}}{4\pi ({\left({r-r_{0})}^{2}+{\left(z_{0}+z\right)}^{2}\right)}^{\frac{1}{2}}}$$

The velocity ***U***=(*U*_r_,*U*_z_) is the vector sum of the spatial gradients of each velocity potential, and its expression is

(14) $$U_{r}=\frac{d\Phi_{1}}{\mathrm{dr}}+\frac{d\Phi_{2}}{\mathrm{dr}}$$

(15) $$U_{z}=\frac{d\Phi_{1}}{\mathrm{dz}}+\frac{d\Phi_{2}}{\mathrm{dz}}$$

(16) $$U_{r}=R_{b}^{2}{\dot{R}}_{b}\left\{\frac{r-r_{0}}{{\left[({r-r_{0})}^{2}+{\left(z_{0}-z\right)}^{2}\right]}^{\frac{3}{2}}}+\frac{r-r_{0}}{{\left[({r-r_{0})}^{2}+({z_{0}+z)}^{2}\right]}^{\frac{3}{2}}}\right\}$$

(17) $$U_{z}=R_{b}^{2}{\dot{R}}_{b}\left\{\frac{-\left(z_{0}-z\right)}{{\left[({r-r_{0})}^{2}+{\left(z_{0}-z\right)}^{2}\right]}^{\frac{3}{2}}}+\frac{z_{0}+z}{{\left[({r-r_{0})}^{2}+({z_{0}+z)}^{2}\right]}^{\frac{3}{2}}}\right\}$$

For double cavitation bubbles, in the theoretical analysis, only the case where the spacing between the cavitation bubbles is large is considered. Therefore, the interaction between the cavitation bubbles is not taken into account, that is, it is assumed that the interaction term between the double cavitation bubbles does not exist. Then, for cavitation bubbles 1 and 2, the Keller-Miksis bubble dynamics equation is used for solution:

(18) $$\left(1-\frac{{\dot{R}}_{1}}{c}\right)R_{1}{\ddot{R}}_{1}+\left(\frac{3}{2}-\frac{{\dot{R}}_{1}}{2c}\right){\dot{R}}_{1}^{2}=\left(1+\frac{{\dot{R}}_{1}}{c}\right)\frac{P_{R1}-p_{0}}{\rho }+\frac{R_{1}}{\rho c}\frac{d\left(P_{R1}-p_{0}\right)}{\mathrm{dt}}$$

(19) $$\left(1-\frac{{\dot{R}}_{2}}{c}\right)R_{2}{\ddot{R}}_{2}+\left(\frac{3}{2}-\frac{{\dot{R}}_{2}}{2c}\right){\dot{R}}_{2}^{2}=\left(1+\frac{{\dot{R}}_{2}}{c}\right)\frac{P_{R2}-p_{0}}{\rho }+\frac{R_{2}}{\rho c}\frac{d\left(P_{R2}-p_{0}\right)}{\mathrm{dt}}$$

Among them, the expressions of *P*_R1_ and *P*_R2_ are as follows:

(21) $$P_{R1}=\left(p_{0}-p_{\nu }+\frac{2\sigma }{R_{0}}\right){\left(\frac{R_{0}^{3}-bR_{0}^{3}}{R^{3}-bR_{0}^{3}}\right)}^{k}-\frac{2\sigma }{R}-\frac{4\mu \dot{R}}{R}+p_{\nu }$$

(22) $$P_{R2}=\left(p_{0}-p_{\nu }+\frac{2\sigma }{R_{0}}\right){\left(\frac{R_{0}^{3}-bR_{0}^{3}}{{R_{2}}^{3}-bR_{0}^{3}}\right)}^{k}-\frac{2\sigma }{R_{2}}-\frac{4\mu {\dot{R}}_{2}}{R_{2}}+p_{\nu }$$

For the particles in the flow field, the main forces acting on them are the fluid drag force *F*_drag_, the flow inertia force *F*_inertia_, the additional mass force *F*_add_, the gravitational force and the buoyant force combined force *F*_b_. The expressions are as follows [49]:

(23) $$F_{drag}=\frac{1}{2}C_{d}\pi R_{p}^{2}\rho_{l}\|U-v_{p}\|\left(U-v_{p}\right)$$

(24) $$F_{\mathrm{inertia}}=\frac{4}{3}\pi R_{p}^{3}\rho_{l}\frac{DU}{Dt}$$

(25) $$F_{add}=\frac{4}{3}\pi R_{p}^{3}C_{M}\rho_{l}\left(\frac{DU}{Dt}-\frac{dv_{p}}{dt}\right)$$

(26) $$F_{b}=\frac{4}{3}\pi R_{p}^{3}\left(\rho_{p}-\rho_{l}\right)g$$

For the force acting on the particles, according to Newton's second law, obtained by:

(27) $$F_{drag}+F_{inertia}+F_{add}+F_{b}=\frac{4}{3}\pi R_{p}^{3}\rho_{p}a_{p}$$

Among them, ***U*** represents the flow field velocity at the particle center position when there are no particles, and *v*_p_ is the velocity of the particle's center. D***U* /** D*t* represents the derivative of ***U***, *ρ*_l_ represents the density of the fluid, *ρ*_p_ represents the density of the particle, *C*_M_ represents the additional mass coefficient, *C*_D_ represents the drag coefficient (taking 0.47[50]), and *a*_p_ represents the particle's acceleration. In addition, the time needs to be normalized. The dimensionless parameter T* = *t* **/** *T*_all_ is defined, where t represents any given time and Tall represents the time of the first cycle of the cavitation bubble expansion and collapse. For a double cavitation bubble, it represents the first cycle time of the smaller cavitation bubble.

**Fig. 5:**
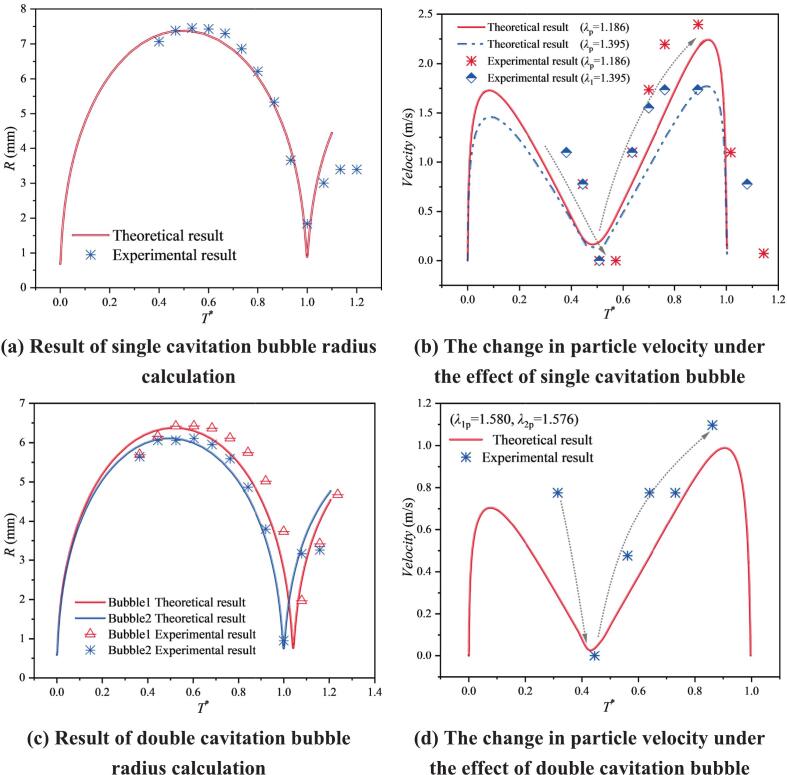
Comparative analysis of theoretical results and experimental results.

In the theoretical analysis, the particle problem under the action of a single cavitation bubble was first examined based on the force balance acting on the particle. Fig. 5(a) compares the experimentally measured cavitation bubble radius with the theoretical prediction. For spark-discharge-generated cavitation bubbles, a luminous emission occurs at the early stage, which prevents clear visualization of the evolution during 0–0.3T*; therefore, only the subsequent stage was captured. The comparison shows that the radius evolution agrees well overall. Fig. 5(b) presents the velocity histories of two particles located at different positions. A preliminary observation is that the maximum particle velocity depends on the distance from the cavitation bubble: a larger distance leads to a smaller maximum velocity. Furthermore, Fig. 5(d) considers the particle velocity for a particle located between an unequal double cavitation bubble. The experimental results show a reasonable agreement with the theoretical analysis over part of the range. Under the superposed influence of the two cavitation bubbles, the maximum particle velocity is clearly lower than that obtained under the action of a single cavitation bubble.

The discrepancies between the experimental measurements and theoretical predictions mainly arise from two factors. First, the theoretical model neglects the interaction between the cavitation bubbles in the derivation. In the experiments, the inter-bubble spacing is *λ*_12_ = 1.619, which falls within an intermediate-distance regime; although the coupling is relatively weak, it is still present. Such residual coupling can modify the local flow evolution rate and momentum-exchange process, leading to deviations of the experimental response from the idealized model. Second, the theoretical calculation does not account for the time-lag effect in particle motion. In the experiments, particles have a finite response time due to viscous drag, and the velocity does not build up instantaneously after the force is applied. This leads to a phase lag in time, i.e., the theoretical curves appear advanced relative to the experimental results. Meanwhile, inter-bubble coupling and the enhancement of the actual flow field may increase the local acceleration intensity, making the experimental peak velocity higher than the theoretical prediction based solely on the single-bubble approximation, which results in an underestimation of the peak amplitude by the theory.

### 3.2 Applicability range of the far-field theoretical model

To provide a comparable and quantitative description of the spacing in a double cavitation bubble system, the dimensionless inter-bubble distance *λ*_12_ is used in this study. Eqs. (18), (19) constitute the far-field theoretical model. Because their derivation relies on the assumption of sufficiently large inter-bubble separation, a reproducible applicability boundary must be specified. In a fully symmetric cavitation bubble system in a free field, the net force of the system cancels out due to symmetry. Therefore, a symmetric half-domain formulation is adopted, and the time-integrated impulse of a single cavitation bubble in a specified direction, extracted from the numerical simulation, is used as the evaluation metric.

To quantify the influence of double cavitation bubble interaction on the impulse of a single cavitation bubble, the half-domain impulse of a single cavitation bubble is taken as the baseline, i.e., *I*_0_ = *I*_single_. A dimensionless impulse proximity coefficient *k* is defined as

(28) $$k=\frac{||I\left(\lambda \right)|-|I_{0}||}{|I_{0}|}$$

where *I*(λ) denotes the impulse of one cavitation bubble in the double-bubble case at a dimensionless spacing *λ*. A smaller k indicates a weaker influence of inter-bubble interaction on this metric, i.e., a smaller interaction effect between the two cavitation bubbles. In this study, *k* ≤ 0.10 (i.e., a relative difference no greater than 10%) is adopted as the criterion for entering the far-field regime. Accordingly, the critical far-field dimensionless spacing is defined as

(29) $$\lambda_{\mathrm{cr}}=\min\left\{\lambda \;:\;k\leq 0.10\right\}$$

A zonal strategy of “far-field analytical model–near-field numerical model” is adopted in this work. As shown in the Table 2, when *λ*_12_ = 2.013, *k* ≤ 0.10, satisfying the far-field criterion; therefore, Eqs. (18), (19) can be used as a far-field approximation model for quantitative prediction and mechanistic interpretation. In addition, observed that 0.10 ≤ *k* ≤ 1.60 remains within an acceptable range; accordingly, 1.4 < *λ*_12_ < 2.0 is defined as an intermediate-distance regime, where the theoretical model can still provide predictive calculations but may involve non-negligible errors and thus requires correction using numerical results. As k increases further, cavitation bubble–cavitation bubble interaction is significantly intensified. Strong near-field coupling amplifies higher-order interactions and enhances non-spherical interface effects, causing the assumptions of the far-field approximation to no longer be strictly valid. Therefore, numerical simulations are emphasized in this interval, and *λ*_12_ < 1.4 is defined as the strong-coupling regime.

**Table 2:** Impulse results and the corresponding *k* values for all cases.

	*λ*_12_	*I*(λ)	*k*
Case2	0.651	9.36 × 10^-3^	5.367
Case3	1.043	5.79 × 10^-3^	2.939
Case4	1.346	3.76 × 10^-3^	1.558
Case5	1.813	2.21 × 10^-3^	0.503
Case0	2.013	1.61 × 10^-3^	0.095

### 3.3 Phenomenon analysis

During the early expansion stage of a cavitation bubble, spark-discharge generation is accompanied by a luminous emission, which prevents the early-time evolution of the cavitation bubble from being captured in the experiments. In addition, during the collapse stage, the cavitation bubble may undergo a non-spherical collapse that occasionally envelops the particles, making it impossible to track the subsequent particle velocity in the later stage of the analysis. Therefore, in this study, the experimental results are reported starting from the stage when the cavitation bubble size is close to its maximum. The particle-motion analysis is also conducted from this instant and terminates after the cavitation bubble completes one collapse event.

#### 3.3.1 Experimental analysis of particle groups under the action of single cavitation bubble

Considering that a particle group contains a large number of particles, it is impractical to statistically track the velocity histories of all particles. Therefore, three representative particles that were easy to identify were selected. By recording particle positions at different instants during the cavitation bubble evolution using a high-speed camera, the velocities of these representative particles were obtained. During high-speed imaging, a ruler with a known length was introduced for spatial calibration. The physical length represented by a single pixel was determined from the ratio between the ruler’s actual length and the corresponding pixel count in the image. ImageJ was then used to measure the target size and displacement in pixels, which were converted into physical values, enabling accurate parameter acquisition and quantitative analysis. Particle velocity was calculated from the measured displacement and the corresponding time interval.

**Fig. 6:**
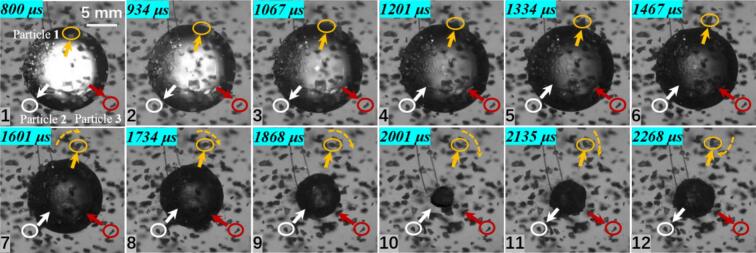
Experimental results of single cavitation bubble and particle group interaction (*λ*_11p_ = 0.903; *λ*_12p_ = 1.186; *λ*_13p_ = 1.395; The yellow circle represents particle 1, the white circle represents particle 2, and the red circle represents particle 3. The direction of the arrow indicates the movement direction of the particles).

As shown in Fig. 6, frame-by-frame measurements indicate that, at a dimensionless parameter *λ*_11p_ = 0.903, Particle 1 reaches a maximum velocity of 6.253 m/s. In addition, when *λ*_11p_ < 1, the particle is strongly influenced by its relatively large early-stage velocity. As a result, in the late stage after cavitation bubble collapse, it does not move toward the bubble center with the bubble contraction; instead, it continues to move away from the bubble center due to inertia. The particles used in the experiments were silicon carbid, with completely irregular shapes. An additional observation was made during particle tracking: before 1467 μs, Particle 1 mainly exhibited motion away from the bubble center, whereas after 1601 μs, Particle 1 showed not only outward translation but also self-rotation. From the perspective of radiation-force attenuation, an irregular particle experiences different magnitudes of radiation pressure at its two ends. The side closer to the cavitation bubble is subjected to a larger radiation pressure, resulting in stronger acceleration, while the far side experiences weaker radiation pressure and thus weaker acceleration. This acceleration imbalance can induce particle rotation. In the numerical-simulation section, this phenomenon is further interpreted from the flow-field characteristics.

When the dimensionless distance to the bubble center increases to 1.186 (Particle 2), the particle moves away from the bubble center during the cavitation bubble expansion stage and moves toward the bubble center during collapse. The maximum velocity of Particle 2 is 2.396 m/s, and no rotation was observed. With a further increase in the dimensionless distance to 1.395 (Particle 3), the maximum velocity decreases to 1.734 m/s, and the motion pattern remains consistent with that of Particle 2. Based on these particle motions at three different distances, the maximum particle velocity decreases markedly with increasing dimensionless distance. This trend is consistent with the attenuation of radiation pressure, further confirming that, in a free field, the cavitation bubble influences particle groups through radiation pressure during both expansion and collapse.

#### 3.3.2 Experimental analysis of particle groups under the action of double cavitation bubbles

**Fig. 7:**
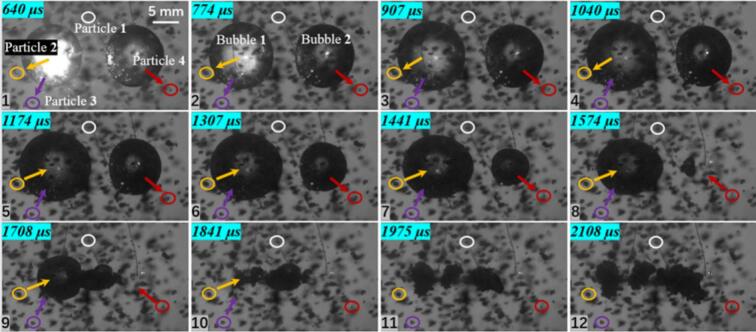
Experimental results of double cavitation bubble(*λ*_12_ = 1.259) and particle group interaction (*λ*_11p_ = 0.903, *λ*_21p_ = 1.975; *λ*_12p_ = 1.103; *λ*_13p_ = 1.395; *λ*_24p_ = 1.097; The white circle represents particle 1, the yellow circle represents particle 2, the purple circle represents particle 3, and the red circle represents particle 4. The direction of the arrow indicates the movement direction of the particles).

As the number of cavitation bubbles increases, a double cavitation bubble in a free field can exhibit a face-to-face collapse. Under this condition, the velocity characteristics of particle groups differ markedly from those in the single cavitation bubble case. As shown in Fig. 7, the inter-bubble spacing is *λ*_12_ = 1.259. The double cavitation bubble undergoes a face-to-face collapse, and a pair of opposing jets appears at a later stage. In the experiments, the overall dynamics of the particle groups could not be captured unambiguously; therefore, velocity analyses were performed for particles located at several specific positions.

For Particle 1, located between the two cavitation bubbles, the measured maximum velocity is 2.327 m/s. This particle is influenced by both cavitation bubbles. Moreover, the experiments indicate that cavitation bubble 2 collapses earlier, resulting in a velocity direction for Particle 1 that is complex and irregular. The maximum velocities of Particle 2 and Particle 3 are 1.734 m/s and 1.097 m/s, respectively. The reduction in velocity is attributed to the decrease in the dimensionless distance. In addition, during both expansion and collapse, Particles 2 and 3 exhibit motions away from and toward the center of cavitation bubble 1, suggesting that particles at these positions are primarily governed by cavitation bubble 1. Particle 4, located on the side of cavitation bubble 2, reaches a maximum velocity of 4.387 m/s. Experimental observations indicate that the premature collapse of cavitation bubble 2 is the main reason for the sharp increase in the velocity of Particle 4.

**Fig. 8:**
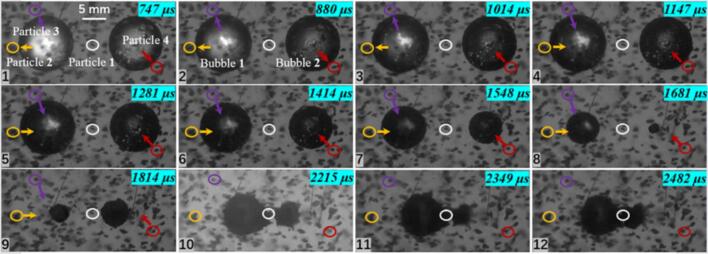
Experimental results of double cavitation bubble(*λ*_12_ = 1.6619) and particle group interaction (*λ*_11p_ = 1.580, *λ*_21p_ = 1.576; *λ*_12p_ = 1.241; *λ*_13p_ = 1.215; *λ*_24p_ = 1.080; The white circle represents particle 1, the yellow circle represents particle 2, the purple circle represents particle 3, and the red circle represents particle 4. The direction of the arrow indicates the movement direction of the particles).

When the inter-bubble spacing increases to 1.619, as shown in Fig. 8, the maximum velocity of Particle 1, located on the line connecting the two bubble centers, decreases to only 0.776 m/s. Particles 2 and 3, located to the left of cavitation bubble 1, reach maximum velocities of 2.453 m/s and 1.734 m/s, respectively. During the expansion stage, both particles move away from the center of cavitation bubble 1, whereas during collapse they move toward the center of cavitation bubble 1, indicating that their motion is primarily governed by cavitation bubble 1. Particle 4, located at the lower-right side of cavitation bubble 2, reaches a maximum velocity of 2.148 m/s and is mainly influenced by the radiation pressure generated during the expansion and collapse of cavitation bubble 2.

**Fig. 9:**
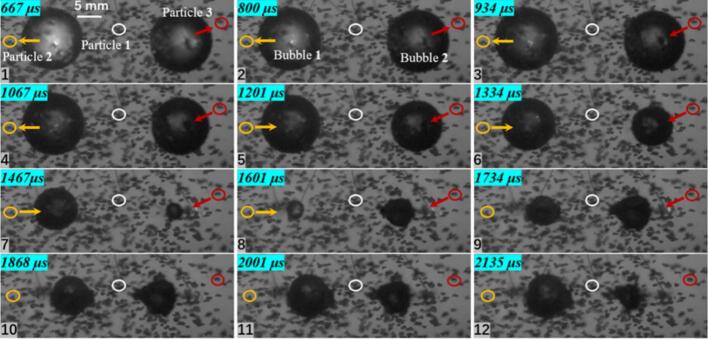
Experimental results of double cavitation bubble(*λ*_12_ = 2.108) and particle group interaction (*λ*_11p_ = 2.138, *λ*_21p_ = 2.102; *λ*_12p_ = 1.338; *λ*_13p_ = 1.436; The white circle represents particle 1, the yellow circle represents particle 2, and the red circle represents particle 3. The direction of the arrow indicates the movement direction of the particles).

Furthermore, when the inter-bubble spacing increases to 2.108, as shown in Fig. 9, the maximum velocities of Particle 2 and Particle 3 are 1.551 m/s and 1.734 m/s, respectively. In this case, the particles are influenced only by their neighboring cavitation bubbles, and the particle located in the intermediate region exhibits almost no noticeable displacement.

#### 3.3.3 Experimental analysis of particle groups under the action of three cavitation bubbles

**Fig. 10:**
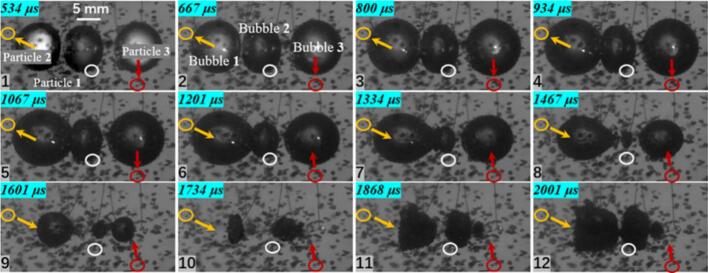
Experimental results of three cavitation bubble (*λ*_12_ = 0.860, *λ*_23_ = 1.125) and particle group interaction (*λ*_21p_ = 1.012; *λ*_12p_ = 1.170; *λ*_33p_ = 1.319; The white circle represents particle 1, the yellow circle represents particle 2, and the red circle represents particle 3. The direction of the arrow indicates the movement direction of the particles).

In the three cavitation bubble case, three representative particles were selected, as shown in Fig. 10. Particle 1 is located beneath the central cavitation bubble 2. During collapse, this region becomes the collapse center of the three cavitation bubble system, where cavitation bubbles 1 and 3 both collapse toward cavitation bubble 2. Therefore, the motion of the particle at this location is expected to differ from that of Particle 2 and Particle 3. Measurements from the experiments show that Particle 1 reaches a maximum velocity of 3.468 m/s, whereas Particle 2, located to the left of cavitation bubble 1, and Particle 3, located directly beneath cavitation bubble 3, reach maximum velocities of 1.734 m/s and 2.327 m/s, respectively, showing a clear difference from the peak velocity of Particle 1. In this configuration, Particles 2 and 3 are still primarily governed by their neighboring cavitation bubbles, and their velocity directions exhibit a clear and regular variation pattern.

**Fig. 11:**
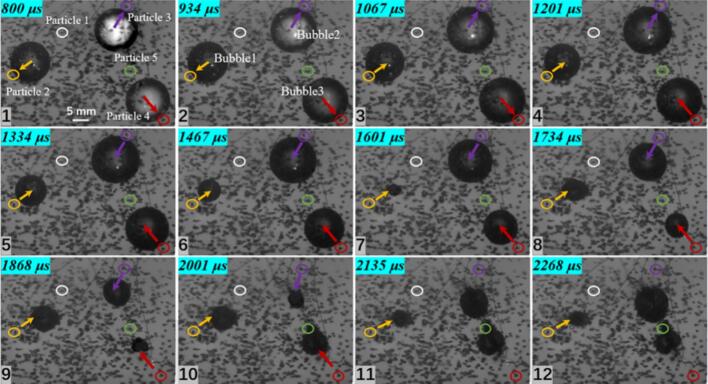
Experimental results of three cavitation bubble (*λ*_12_ = 2.101, *λ*_13_ = 1.671, *λ*_23_ = 2.882) and particle group interactio (*λ*_21p_ = 2.114, *λ*_11p_ = 2.351; *λ*_22p_ = 1.153; *λ*_13p_ = 1.045; *λ*_34p_ = 1.013; *λ*_15p_ = 1.795, *λ*_35p_ = 1.523; The white circle represents particle 1, the yellow circle represents particle 2, the purple circle represents particle 3, and the red circle represents particle 4. The direction of the arrow indicates the movement direction of the particles).

A three cavitation bubble system can be arranged not only in a collinear configuration but also in a triangular configuration, as shown in Fig. 11. Particle 1, located between cavitation bubbles 1 and 2, and Particle 5, located between cavitation bubbles 2 and 3, both exhibit a measured maximum velocity of 1.097 m/s. In contrast, the maximum velocities of Particles 2, 3, and 4 are 2.453 m/s, 3.468 m/s, and 1.734 m/s, respectively. The spacing between the cavitation bubbles affects both the motion direction and the velocity magnitude of nearby particles.

By varying the number of cavitation bubbles and the inter-bubble parameters, it is found that particle velocity decreases markedly as the distance between the cavitation bubble and the particle increases. For both double cavitation bubble and three cavitation bubble configurations, particles located in the region between cavitation bubbles exhibit velocity magnitudes and direction patterns that differ substantially from those of particles primarily influenced by a single cavitation bubble. These distinct velocity characteristics of particle groups across different regions warrant further investigation and analysis.

### 3.4 Numerical simulation study

Due to the limitations of experimental imaging, only the velocities of particles outside the cavitation bubble can be observed. Therefore, capturing the overall velocity characteristics of particle groups under the action of a cavitation bubble cannot rely on experimental analysis alone. In addition, under strong inter-bubble coupling, theoretical analysis has intrinsic limitations. For the near-distance and intermediate-distance cases, detailed numerical simulations were also performed. From the two perspectives of velocity direction and velocity magnitude, the velocity characteristics of particle groups in a free field are discussed.

In this study, a pressure-based transient solver was employed to perform transient simulations of gas–liquid two-phase flow. The liquid phase was water, and the gas phase was water vapor. The gas–liquid interface was captured using the VOF (Volume of Fluid) model. The VOF model assumes that the two phases share the same velocity and pressure fields, and the interface position is tracked via the transport equation of the gas-phase volume fraction, *α*_v_. Phase-change mass transfer was not considered in this work; the gas-phase region (i.e., the bubble) was prescribed by the initial condition, and its subsequent evolution was governed by flow inertia and interfacial dynamics.

Let *α*_v_ denote the gas-phase volume fraction. Without phase-change mass transfer, the volume-fraction transport equation is given by:

(30) $$\frac{\partial \alpha_{v}}{\partial t}+\nabla \cdot \left(\alpha_{v}u\right)=0$$

Subject to the following constraint:

(31) $$\alpha_{l}\;+\;\alpha_{v}=1$$

The mixture density is weighted by the volume fractions:

(32) $$\rho =\alpha_{v}\rho_{v}+\left(1-\alpha_{v}\right)\rho_{l}$$

Momentum equation:

(33) $$\frac{\partial \left(\rho u\right)}{\partial t}+\nabla \cdot \left(\rho uu\right)=-\nabla p+\nabla \cdot \tau +\rho g+S_{M}$$

where *ρ* is the mixture density, *ρ*_v_ is the vapor-phase density, *ρ*_l_ is the liquid-phase density, τ is the viscous stress tensor, and $S_{M}$ represents the additional momentum source term from the discrete phase to the continuous phase.

(34) $$\tau =\mu \left[\nabla u+{\left(\nabla u\right)}^{T}-\frac{2}{3}\left(\nabla \cdot u\right)I\right]$$

The particle phase is treated using the DPM approach and tracked in a Lagrangian framework. The particle dynamical equation is written as [51]:

(35) $$m_{p}\frac{du_{p}}{\mathrm{dt}}=F_{\mathrm{drag}}+F_{\mathrm{intertia}}+F_{\mathrm{add}}+F_{b}$$

In this study, heat exchange between the solid and liquid phases is neglected, and only the exchange of momentum and turbulent kinetic energy is considered. The two-phase coupling is realized by iteratively solving the governing equations for the liquid phase and the discrete phase. Particle momentum exchange occurs along their trajectories within each fluid-phase control volume, and can be expressed as [52]:

(36) $$S_{M}=\sum \left(F_{\mathrm{drag}}+F_{\mathrm{intertia}}+F_{\mathrm{add}}+F_{b}\right)M_{p}\Delta t$$

where $M_{p}$ is the particle mass flow rate, and Δt is the time step.

A pressure-based transient solver was used, and the pressure–velocity coupling was handled using the Coupled scheme. For spatial discretization, PRESTO! was adopted for pressure, Second Order Upwind for the momentum equations, and a Compressive scheme for the VOF volume-fraction equation. Time discretization was performed using the First Order Implicit method. The computational domain size was 90 mm × 90 mm × 90 mm. All boundaries in the free field were specified as pressure outlet, with the reference pressure set to 101325 Pa.

A structured hexahedral mesh was employe. A grid-independence study was conducted using an initial bubble radius of *R*_0_ = 0.5 mm and an initial pressure of *p*(*R*_0_) = 98 MPa, by comparing the maximum bubble radius *R*_max_ under five mesh-resolution schemes in the diameter direction. The results are summarized in Table 3: when the number of cells across the bubble diameter exceeded 50, *R*_max_ became nearly insensitive to further refinement. Considering both accuracy and computational cost, Scheme 4 (with 60 cells across the bubble diameter) was adopted in this work, corresponding to a total of approximately 11.6 million cells. The time step was set to Δ*t* = 10^−8^ s [53], [54].

**Table 3:** Grid-independence analysis.

Scheme	Number of cells across the bubble diameter	*R*_max_(mm)
1	30	7.59
2	40	7.43
3	50	7.32
4	60	7.29
5	75	7.31

In this study, the initial size, position, and internal state of the cavitation bubble were specified using MATLAB together with the Patch initialization procedure. First, based on the experimental conditions, the initial internal pressure and geometric scale of the cavitation bubble were determined in MATLAB, with the initial bubble pressure set to *p*(*R*_0_) = 98 MPa and the initial radius set to *R*_0_ = 0.5 mm. A standard initialization was then applied to the entire domain such that the initial field was a pure liquid phase (*α*_l_ = 1and *α*_v_ = 0), with a uniform ambient pressure of 101325 Pa. To generate a bubble at a prescribed location, Cell Register was used to define the set of cells occupied by the cavitation bubble. A corresponding register was created according to a spherical geometric criterion (one register for a single bubble; multiple registers were created separately for multi-bubble cases). Based on these registers, the Patch operation was applied to impose the initial field variables within each registered region: the gas-phase volume fraction was assigned as *α*_v_ = 1 (indicating that the cavitation bubble interior is fully occupied by the gas phase), and the pressure in the same region was set to 98 MPa. In this way, cavitation bubbles with the prescribed radius, center position, and initial internal pressure were obtained. Finally, the initialization was verified using contours of volume fraction and pressure to confirm that the bubble geometry, positional relation, and the high-pressure region inside the bubble were all consistent with the prescribed settings.

For numerical simulations of cavitation bubble–particle group coupling in a free field, two key aspects are involved: the evolution of the cavitation bubble and the capture of particle trajectories. The VOF multiphase model was adopted to resolve the interfacial evolution of the cavitation bubble. To model the interaction between the fluid and particle groups, the DPM model (Euler–Lagrange approach) was employed. The computational domain was 90 mm × 90 mm × 90 mm. Based on the particle distribution observed in the experiments, particle groups were initialized within the computational domain. Repeated experiments indicated that the particle size ranged from 26 to 40 mesh (0.43–0.71 mm). According to this size range, the particles were classified into three size groups, with a total of 6000 particles. Considering the size of the dataset, we wrote a Python script to generate the particle group dataset and initialize the particle groups, enabling the implementation of 6000 particles (0.43–0.71 mm) to fill the computational domain. Particle tracking was performed with two-way coupling between the discrete phase and the continuous phase. The DPM iteration interval was set to 10 [55], and the maximum number of tracking steps was set to 500.

**Fig. 12:**
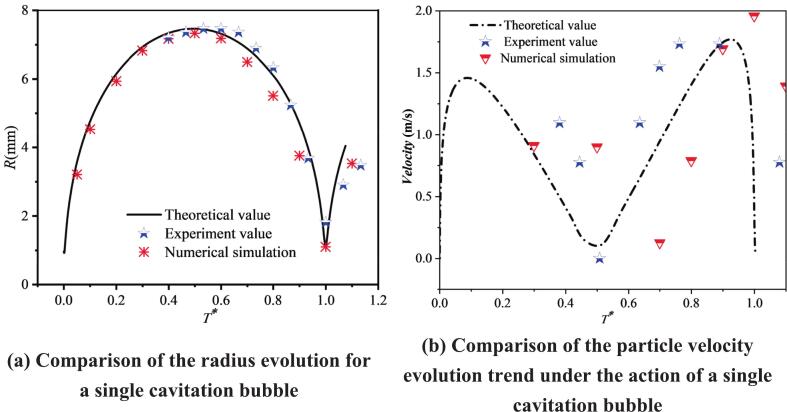
Comparison among experiment, theory, and numerical simulation.

The reliability of the numerical results is critical for evaluating the velocity characteristics of particle groups. Therefore, the cavitation bubble radius was selected as the key validation metric. By comparing the single cavitation bubble radius obtained from the numerical simulation with the corresponding experimental measurements and theoretical predictions, the reliability of the numerical model was assessed. As shown in Fig. 12, the radius evolution predicted by the numerical simulation agrees well overall with both the theoretical analysis and the experimental results. In addition, it is observed that the three approaches can also capture the particle-velocity characteristics reasonably well, indicating that the numerical simulation can reliably reproduce the cavitation bubble dynamics as well as the coupled dynamics associated with cavitation bubble–particle interactions.

#### 3.4.1 Velocity-direction characteristics of particle groups under different cavitation-bubble conditions

**Fig. 13:**
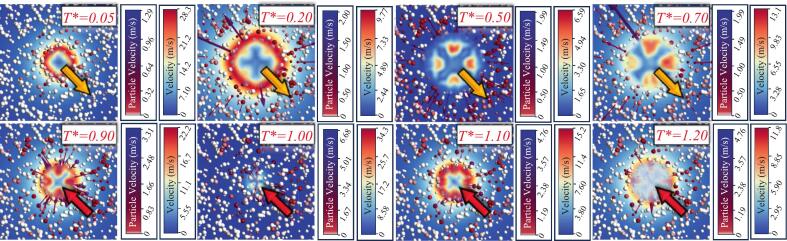
Numerical simulation results of a single cavitation bubble interacting with particle groups.

During the interaction between a single cavitation bubble and particle groups, the particle velocity directions exhibit pronounced changes as the cavitation bubble expands and collapses, as shown in Fig. 13. The initial stage (Frames 1–3) corresponds to the cavitation bubble expansion phase. In the figure, the arrow direction indicates the particle velocity direction. During this phase, the velocities radiate outward from the cavitation bubble center. The arrow length and color represent the velocity magnitude. It is observed that particles closer to the cavitation bubble center have markedly higher velocities than those farther away. This phenomenon is consistent with the experimentally observed trend that particle velocity decreases with increasing distance. This outward-radiating velocity attenuation trend is also fundamentally related to the rotation of irregular particles observed in the experiments in Fig. 6: for an irregular particle, the velocity difference between the side closer to the cavitation bubble and the side farther away can generate a torque, causing particle rotation. This is a special observation identified in this study. However, because the primary objective here is to characterize the coupling dynamics between cavitation bubbles and particle groups, spherical particles were adopted in the numerical simulations, which to some extent neglects the influence of particle irresgularity on such rotation-related phenomena.

In addition, at 1.0T*, the velocity field of the particle groups indicates that the maximum particle velocity reaches 6.68 m/s, which is close to the experimentally observed peak velocity of 6.253 m/s for the single cavitation bubble case. The remaining difference is mainly because the particle positions in the experiments and simulations cannot be made exactly identical. This agreement further demonstrates that the numerical simulation can accurately capture the coupling characteristics between the cavitation bubble and particle groups.

In a free field, a single cavitation bubble acts in a manner similar to a point source. However, when the number of cavitation bubbles increases, the newly introduced cavitation bubble inevitably exerts a pronounced influence on the flow field. The following section discusses the interaction between a double cavitation bubble and particle groups in a free field. Four inter-bubble spacings were considered.

**Fig. 14:**
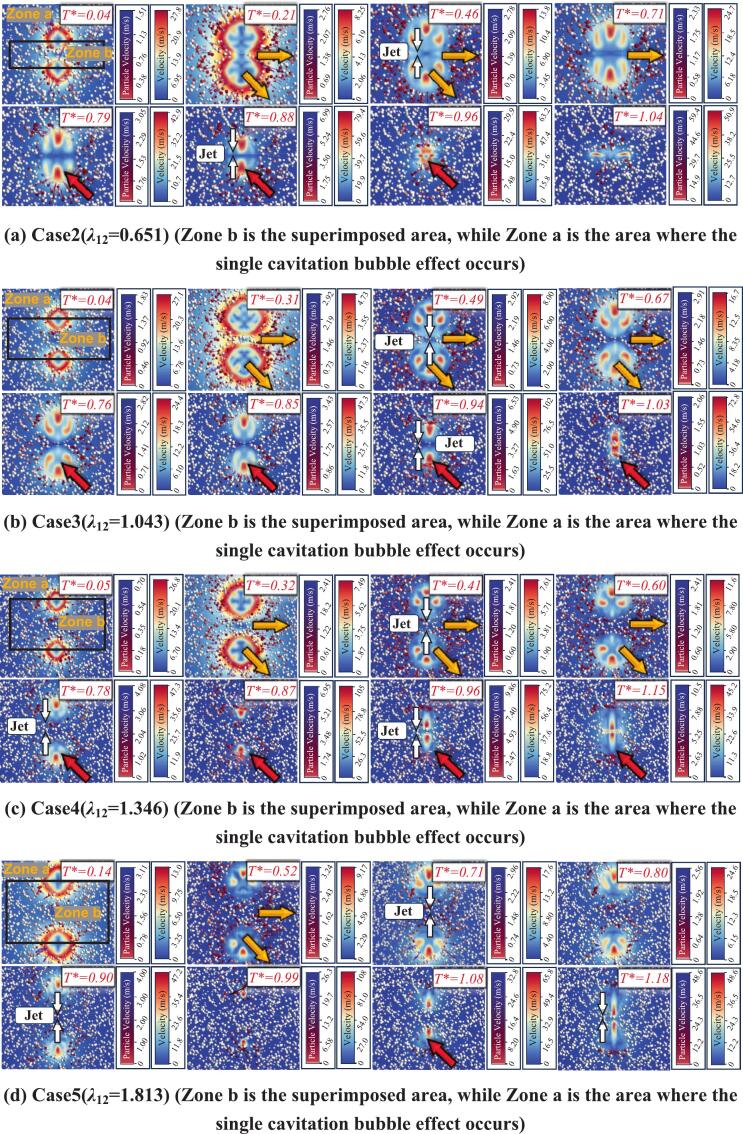
Numerical simulation results of a double cavitation bubble interacting with particle groups.

For the short-spacing double cavitation bubble system, strong interference occurs between the two cavitation bubbles. As shown in Case 2 of Fig. 14 with *λ*_12_ = 0.651, particles in Zone a exhibit a behavior similar to that in the single cavitation bubble case: during the initial expansion stage (Frames 1–3), the particle velocity directions are away from the bubble center. However, due to the small inter-bubble spacing, the superposition region denoted as Zone b in Frame 1 shows a distinct feature. In this region, particle velocity directions point neither toward the center of cavitation bubble 1 nor toward the center of cavitation bubble 2, but are instead almost perpendicular to the line connecting the two bubble centers. This is a consequence of the superposed effect of radiation pressure from the two cavitation bubbles. As also seen in Fig. 14(a), a pronounced face-to-face jet forms between the two cavitation bubbles. At 0.88T*, the jet velocity reaches 79.4 m/s, and the particle-group velocities continue to increase under the action of the microjet. This value is far greater than the particle acceleration induced by a single cavitation bubble, further indicating that microjets generated by inter-bubble interaction can enhance the coupling intensity between cavitation bubbles and particle groups. The emergence of the face-to-face microjet breaks the original flow-field characteristics of the superposition region (Zone b), causing particle velocities in this region to no longer align with the centers of cavitation bubble 1 or cavitation bubble 2, but instead to exhibit the aforementioned direction nearly perpendicular to the center-connecting line. In addition, at 0.21T*, the flow velocity in Inside the cavitation bubble is observed to be very low, and the flow velocity outside the cavitation bubble contour decays gradually outward. Such a flow-field feature leads particle velocity directions to follow the local flow-velocity attenuation direction, resulting in the nearly perpendicular directions relative to the center-connecting line.

When the inter-bubble spacing increases to *λ*_12_ = 1.043 (Case 3 in Fig. 14), the particle velocity-direction characteristics during the expansion stage remain consistent with those in Case 2. During collapse, however, particles in Zone a begin to change their velocity directions from pointing away from the bubble center to pointing toward the bubble center. For particles in Zone b, no clear regularity in the velocity-direction evolution is observed. For example, in Frame 6 of Case 3, particles beneath cavitation bubble 2 exhibit velocity directions that are approximately parallel to the line connecting the two bubble centers. Under the complex influence in the superposition region, the directionality of particle motion becomes less definite. Combined with the flow-field features at 0.31T*, it can be seen that, as the inter-bubble spacing increases, noticeable flow-velocity variations also develop along the center-connecting line within Zone b. This change causes the particle velocity directions in the superposition region to gradually shift from being nearly perpendicular to the center-connecting line toward an outward-radiating pattern from the cavitation bubble centers.

When the inter-bubble spacing further increases to *λ*_12_ = 1.346, the inter-bubble interference continues to weaken. In Zone b, some particles still show velocity directions nearly perpendicular to the center-connecting line, while near the center-connecting line, some particles exhibit almost zero velocity. This implies that the superposed effect at these locations is nearly negligible, and such particles are effectively “isolated” during the expansion stage, as illustrated by the particles in Frames 2–3 of Case 4 in Fig. 14. During collapse, a face-to-face microjet forms, and the resulting jet accelerates particles near the center-connecting line (Frames 5–8 of Case 4 in Fig. 14). The accelerated particle velocities will be discussed in detail in Section 3.2. At this spacing, the particle velocity directions in Zone b become closer to those in Zone a, indicating that the constraining influence of the superposition region on particle motion is weakened.

For Case 5 with λ12 = 1.813, Fig. 14(d) shows that the system behavior becomes similar to the single cavitation bubble case, except that a face-to-face microjet forms during collapse. Due to the larger inter-bubble spacing, the microjet intensity is significantly reduced. Consequently, the particle velocity directions in the superposition region are nearly indistinguishable from those in the single cavitation bubble case, indicating that the constraining influence of inter-bubble superposition has attenuated with increasing distance. This trend also reflects the strong attenuation characteristic of radiation pressure.

For the three cavitation bubble problem, due to the complexity and diversity of spatial arrangements, this study focuses on two representative configurations. The first is a collinear (horizontal) arrangement. Previous studies have shown that the relative magnitude of *λ*_12_ and *λ*_23_ is a key parameter governing the expansion–collapse characteristics of a collinear three cavitation bubble system. Accordingly, Case 6 and Case 7 are discussed for this configuration. The second configuration is a triangular arrangement. Case 8 corresponds to equal pairwise spacings, whereas Case 9 increases one of the pairwise spacings.

**Fig. 15:**
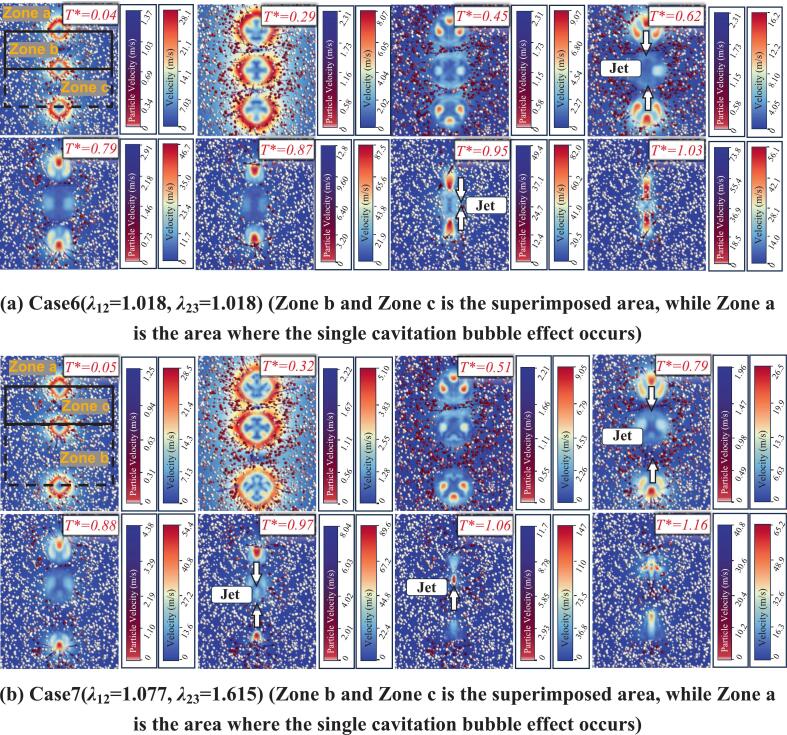
Numerical simulation results of a horizontally arranged three cavitation bubble system interacting with particle groups.

When *λ*_12_ and *λ*_23_ are equal in a three cavitation bubble system, as shown in Case 6 of Fig. 15, Zone b and Zone c correspond to superposition regions formed by the combined influence of neighboring cavitation bubbles. In these two superposition regions, particle velocity directions remain nearly perpendicular to the line connecting the bubble centers. In contrast, particles in Zone a are mainly governed by the nearest cavitation bubble: during the expansion stage they move away from the center of the adjacent cavitation bubble, while during collapse they move toward the adjacent bubble center. This behavior arises because the strong coupling in the superposition regions prevents the flow velocity from decaying along the center-connecting line. Under the superposed action of two nearly equal and oppositely directed radiation pressure contributions, the particle velocity component along the center-connecting line becomes approximately zero, leaving only the force component perpendicular to that direction. Consequently, particles exhibit motion that is almost perpendicular to the center-connecting line, consistent with the superposition region observed in the double cavitation bubble case.

When *λ*_12_ and *λ*_23_ are different, as shown in Case 7 of Fig. 15, Zone b and Zone c are still superposition regions, but the superposition effects differ markedly due to the unequal spacings. In Zone b, because of the larger inter-bubble spacing, the superposition effect is weakened. As observed in Frames 2–3 of Case 7 in Fig. 15, some particles on the right side of Zone b exhibit velocity directions pointing away from the bubble center rather than being perpendicular to the center-connecting line, reflecting the reduced superposition constraint. By contrast, *λ*_23_ is unchanged, so the superposition effect in Zone c is not weakened, and the particle velocity directions there remain perpendicular to the corresponding center-connecting line. During collapse, particles in Zone a point toward the centers of the adjacent cavitation bubbles, similar to the single cavitation bubble behavior. Moreover, on the right side of the superposition region Zone b, particles also show velocity directions pointing toward the center of cavitation bubble 2, as illustrated in Frame 7 of Case 7 in Fig. 15.

**Fig. 16:**
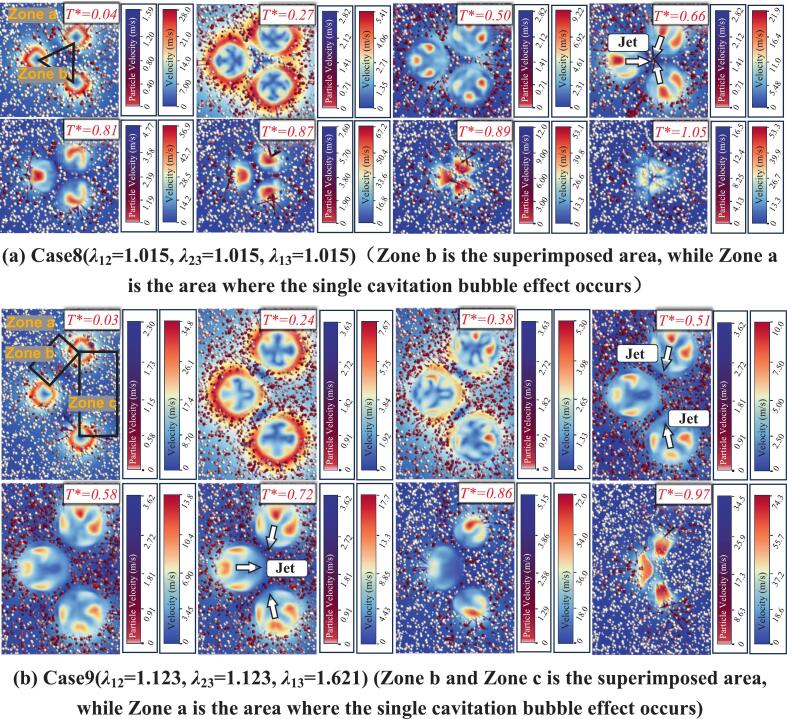
Numerical simulation results of a triangular three cavitation bubble system interacting with particle groups.

For the triangular configuration, Frames 1–8 of Case 8 in Fig. 16 show that, when the pairwise spacings are equal, the particle velocity characteristics under a three cavitation bubble system are broadly similar to those under a single cavitation bubble, and more strongly exhibit collective (group-like) behavior. In Frames 1–4, particles in Zone a move away from the common geometric center of the three cavitation bubbles, rather than away from the center of any individual cavitation bubble. This indicates that the three cavitation bubbles can be approximately treated as an equivalent larger single cavitation bubble. During collapse, some particles can be observed to move toward the common center of the three cavitation bubbles, while others exhibit irregular directions, as shown in Frames 5–8. The flow-field velocity directions also tend to point toward the center of Zone b. In terms of flow velocity magnitude, this configuration produces lower velocities than the collinear arrangement. The maximum jet velocity reaches 67.2 m/s at 0.87T*, implying that the particle dynamic parameters under this condition are correspondingly reduced.

When the spacing between the two cavitation bubbles on the right side is increased, the collective behavior observed in Case 8 is disrupted. As shown in Case 9 of Fig. 16, particles in Zone b and Zone c exhibit velocity directions that are nearly perpendicular to their respective bubble center-connecting lines. During collapse, particles in Zone a still tend to point toward the centers of their neighboring cavitation bubbles rather than toward the common center of the three cavitation bubble system. In this case, the maximum jet velocity reaches 70 m/s, which is significantly higher than that in Case 8. This indicates that the microjet intensity generated by an isosceles triangular configuration is stronger than that generated by an equilateral triangular configuration in a three cavitation bubble system.

#### 3.4.2 Velocity-magnitude characteristics of representative particles in particle groups

The velocity-direction characteristics of particle groups are mainly governed by two factors: the influence of the nearest single cavitation bubble and the superposition effects induced by multiple cavitation bubbles. Accordingly, distinct behaviors are also expected in terms of velocity magnitude. If these mechanisms persist, they may further reveal the complex interaction between multi-cavitation-bubble systems and particle groups. Therefore, this study continues by investigating the velocity magnitudes of particles located at several specially selected positions.

**Fig. 17:**
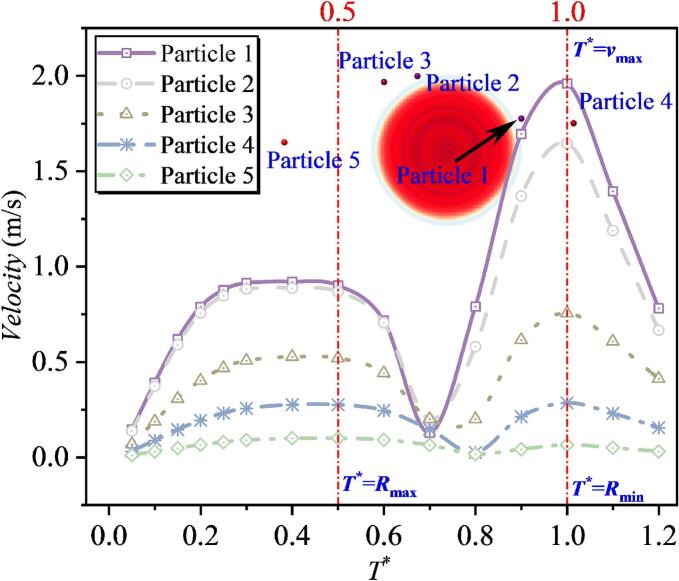
The velocity of particles under the effect of single cavitation bubble (Case 1: *λ*_11p_ = 1.082; *λ*_12p_ = 1.109; *λ*_13p_ = 1.391; *λ*_14p_ = 1.780; *λ*_15p_ = 2.565).

In the numerical simulations for both the single-particle and particle-group cases, five particles at different distances were selected and their velocities were tracked. Since the velocity-direction characteristics have been analyzed in the previous section, this part focuses mainly on the velocity magnitude. As shown in Fig. 17, the overall trend is that, during 0–0.3T*, particle velocity increases. This increase is induced by cavitation bubble expansion. During 0.3T*–0.5T*, particle velocity varies only slightly and remains approximately constant. It is also observed that a smaller dimensionless bubble-to-particle distance *λ*_1ip_ (where *i* denotes the *i*th particle) corresponds to a larger plateau value. After 0.5T*, as the cavitation bubble collapses, the direction of radiation pressure begins to change and the particle force becomes directed toward the bubble center. As a result, particle velocity shows a clear decreasing trend. When the velocity decreases to a certain level, the velocity direction switches from pointing away from the bubble center to pointing toward the bubble center. Subsequently, the velocity increases again, and the particle velocity reaches its maximum at approximately 1.0T*. The dependence of the peak velocity during collapse is similar to that during expansion: a larger *λ*_1ip_ leads to a smaller peak velocity. Among the five particles, the maximum velocity of Particle 1 during collapse is close to 2.0 m/s. By comparing the velocity histories of the five particles over the entire process, it is further found that, for smaller *λ*_1ip_, the difference between the maximum velocity in the expansion stage and that in the collapse stage is larger. For example, for Particle 1, the maximum velocity during expansion is 0.919 m/s, whereas that during collapse is 1.959 m/s, giving a difference of 1.04 m/s. With increasing *λ*_1ip_, this difference gradually decreases. For Particle 4 with *λ*_14p_ = 1.780, the two peak velocities become comparable, and beyond this distance, the peak velocity during collapse becomes smaller than that during expansion. This indicates that particles closer to the cavitation bubble are more strongly affected by the collapse stage, whereas particles farther away are mainly influenced by the expansion stage. In addition, by examining the cavitation bubble shape evolution, it is observed that at T* = 0.5, the cavitation bubble radius reaches its maximum, after which particle velocity decreases initially. At T* = 1.0, the cavitation bubble radius reaches its minimum, and particle velocity attains its maximum.

Introducing cavitation bubble 2 in the double cavitation bubble system modifies the forces acting on particles in the original flow field. Moreover, in a free field, a double cavitation bubble system can generate a face-to-face microjet, and the jet impact is expected to affect particle velocity. Therefore, the following section analyzes particle velocities under the action of a double cavitation bubble for different inter-bubble spacings.

**Fig. 18:**
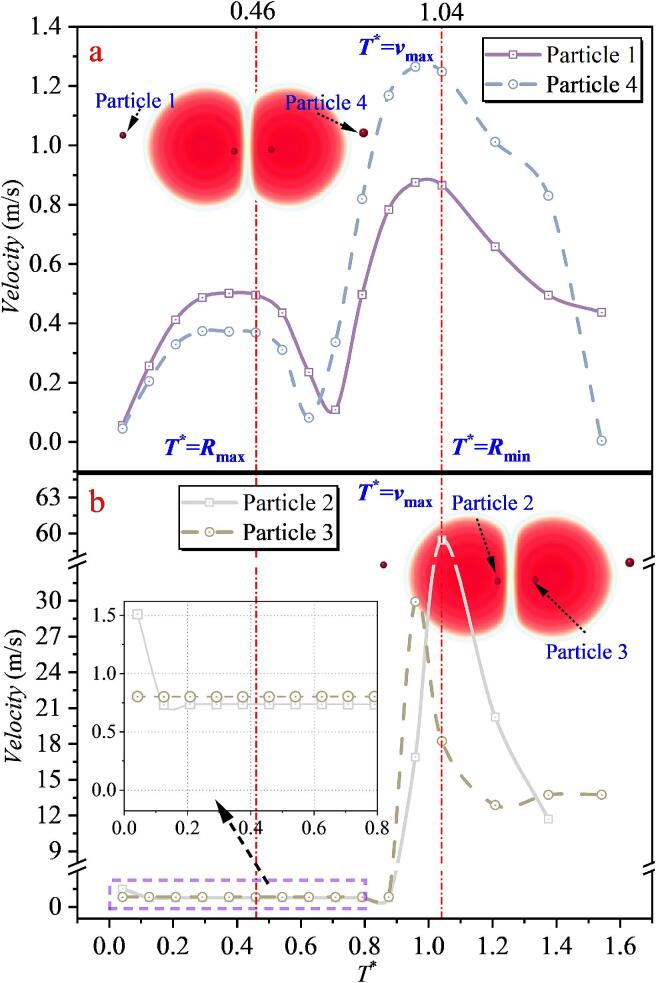
The velocity of particles under the effect of double cavitation bubbles (*λ*_12_ = 0.651) (Case 2: *λ*_11p_ = 1.347; *λ*_12p_ = 0.467, *λ*_22p_ = 0.834; *λ*_13p_ = 1.071, *λ*_23p_ = 0.239; *λ*_24p_ = 1.204).

When the inter-bubble spacing is *λ*_12_ = 0.651, tracking of particle velocities shows that the outermost particles, Particle 1 (far left) and Particle 4 (far right), follow a trend similar to that in the single cavitation bubble case: their velocities first decrease markedly and then increase again. However, the maximum velocities of Particles 1 and 4 throughout the entire process remain below 1.4 m/s. In contrast, the particles located in the intermediate region between the two cavitation bubbles (Particles 2 and 3) exhibit a fundamentally different behavior from that of Particles 1 and 4. As shown in Fig. 18(b), under the superposed radiation pressure of the double cavitation bubble system, the velocities of Particles 2 and 3 remain nearly constant at 0.7–0.8 m/s during 0–0.8T*, without the pronounced variations observed for Particles 1 and 4. After 0.8 T*, the microjet generated between the cavitation bubbles impinges on and accelerates the particles, causing the maximum velocity of Particle 2 to reach 59.433 m/s, while that of Particle 3 reaches 29.913 m/s. Such velocity amplification is far beyond what can be achieved by radiation pressure alone. The difference between the peak velocities of Particles 2 and 3 mainly arises from their random initial distribution. Because a three-dimensional model is adopted, Particle 2 is located nearly at the jet-impact centerline, whereas Particle 3 is offset from that region, leading to the discrepancy in peak values. Combined with the bubble-shape evolution, it is found that at T* = 0.46, the double cavitation bubble radius reaches its maximum, after which the velocities of Particles 1 and 4 decrease and then increase again. At T* = 1.04, the double cavitation bubble radius reaches its minimum, and Particles 1–4 attain their maximum velocities around this instant.

**Fig. 19:**
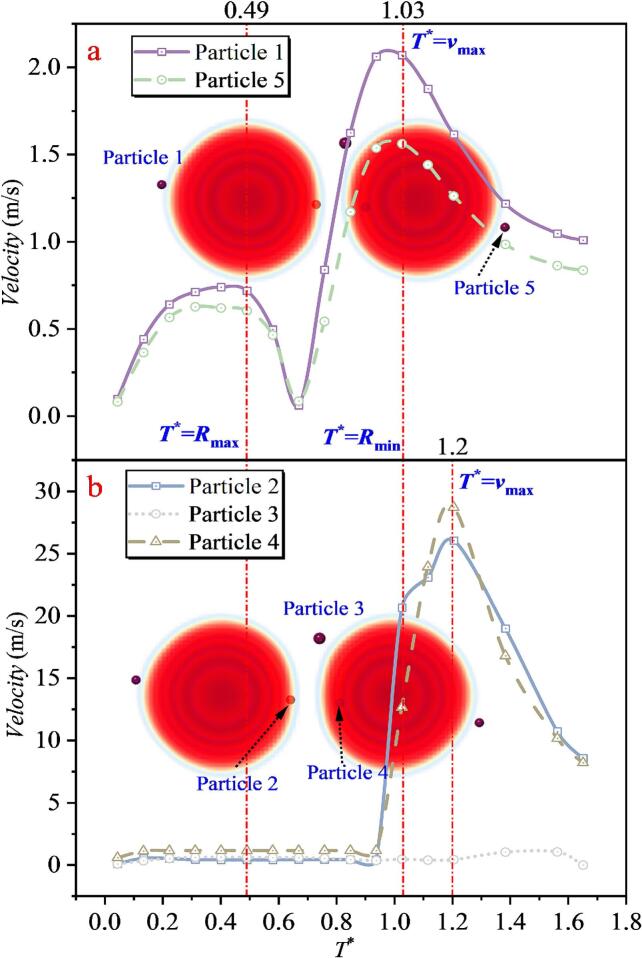
The velocity of particles under the effect of double cavitation bubbles (*λ*_12_ = 1.043) (Case 3: *λ*_11p_ = 1.071; *λ*_12p_ = 0.814, *λ*_22p_ = 1.181; *λ*_13p_ = 1.435, *λ*_23p_ = 1.229; *λ*_14p_ = 1.344, *λ*_24p_ = 0.566; *λ*_25p_ = 1.154).

When the inter-bubble spacing increases to *λ*_12_ = 1.043, as shown in Fig. 19, the velocity evolution of Particle 1 and Particle 5 remains consistent with that in the single cavitation bubble case. For the intermediate region, three particles were selected. Particles 2 and 4 are still located close to the line connecting the bubble centers, and the collapse-induced microjet can accelerate them. However, due to the increased spacing, the maximum velocities of Particles 2 and 4 are reduced to 26.043 m/s and 28.714 m/s, respectively, which are smaller than the peak accelerations in Case 2. In addition, a particle farther away from the center-connecting line (Particle 3) was selected. Although Particle 3 is also located in the intermediate region, its maximum velocity is only 1.041 m/s, which is far lower than those of Particles 2 and 4, and even lower than those of Particles 1 and 5. This sharp reduction is attributed, first, to its initial position being far from the jet core region, and second, to the combined action of the two cavitation bubbles, which makes it difficult for the particle to experience a sustained force in a single direction that would lead to continuous acceleration. By contrast, Particles 1 and 5 are primarily influenced by the radiation pressure of a single nearby cavitation bubble, enabling a sustained force in a consistent direction and thus producing more evident acceleration or deceleration. By correlating these results with the bubble-shape evolution, it is found that at T* = 0.49, the double cavitation bubble radius reaches its maximum. After this instant, the velocities of Particles 1 and 5 decrease and then increase again, whereas the velocity variations of Particles 2, 3, and 4 are not pronounced. At T* = 1.03, the double cavitation bubble radius reaches its minimum. Particles 1 and 5 attain their maximum velocities around this instant, whereas Particles 2 and 4 reach their peak velocities near T* = 1.20. The differences in the timing of peak velocities reflect the direction of momentum transfer in the flow field, namely, from the outer region of the cavitation bubbles (locations of Particles 1 and 5) toward the intermediate region between the two cavitation bubbles (locations of Particles 2 and 4).

**Fig. 20:**
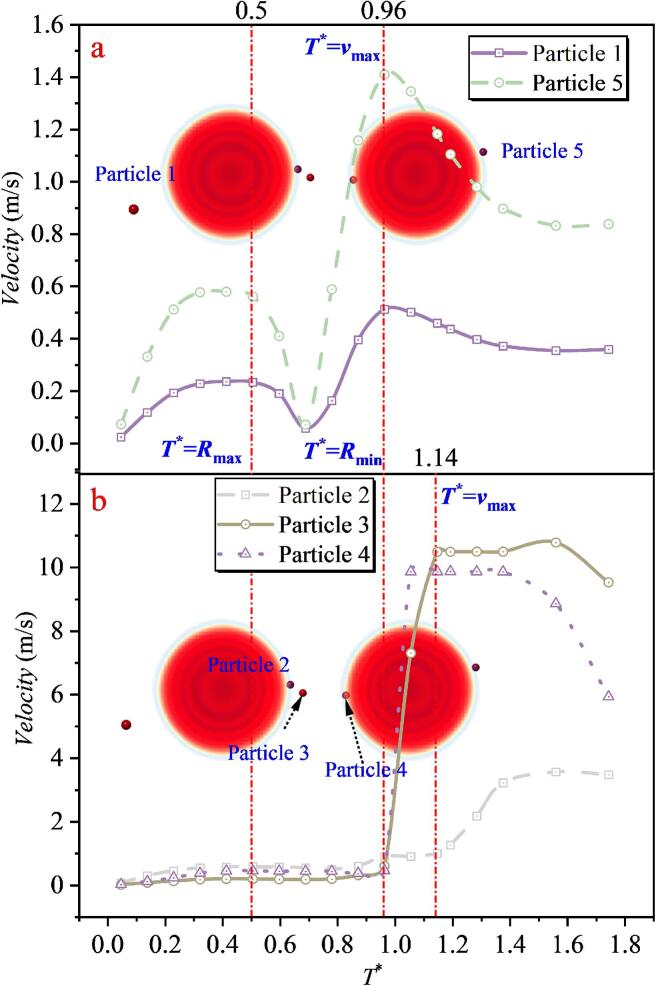
The velocity of particles under the effect of double cavitation bubbles (*λ*_12_ = 1.346) (Case4: *λ*_11p_ = 1.498; *λ*_12p_ = 1.144, λ_22p_ = 1.822; *λ*_13p_ = 1.159, λ_23p_ = 1.528; *λ*_14p_ = 1.195, *λ*_24p_ = 0.901; *λ*_25p_ = 1.195).

With a further increase in the inter-bubble spacing *λ*_12_, as shown in Fig. 20, the velocity-magnitude evolution of Particles 1 and 5 does not change noticeably. However, as the microjet impact weakens, the peak velocities of particles in the intermediate superposition region decrease. The maximum velocities of Particles 3 and 4 reduce to only 10.786 m/s and 9.867 m/s, respectively, which are substantially lower than those in Case 3. The maximum velocity of Particle 2 is further reduced to 3.567 m/s.

By correlating these results with the double cavitation bubble shape evolution, it is found that at T* = 0.50, the double cavitation bubble radius reaches its maximum, after which the velocities of Particles 1 and 5 decrease. The velocities of Particles 2, 3, and 4 exhibit trends similar to those in Case 3, with no pronounced variations. At T* = 0.96, the double cavitation bubble radius reaches its minimum. Particles 1 and 5 attain their maximum velocities around this instant, whereas Particles 3 and 4 reach their peak velocities near T* = 1.14. The differences in the timing of peak velocities again indicate the momentum-transfer direction in the flow field, namely, from the outer region of the cavitation bubbles (locations of Particles 1 and 5) toward the intermediate region between the two cavitation bubbles (locations of Particles 2 and 3). This feature is also observed in Case 3.

**Fig. 21:**
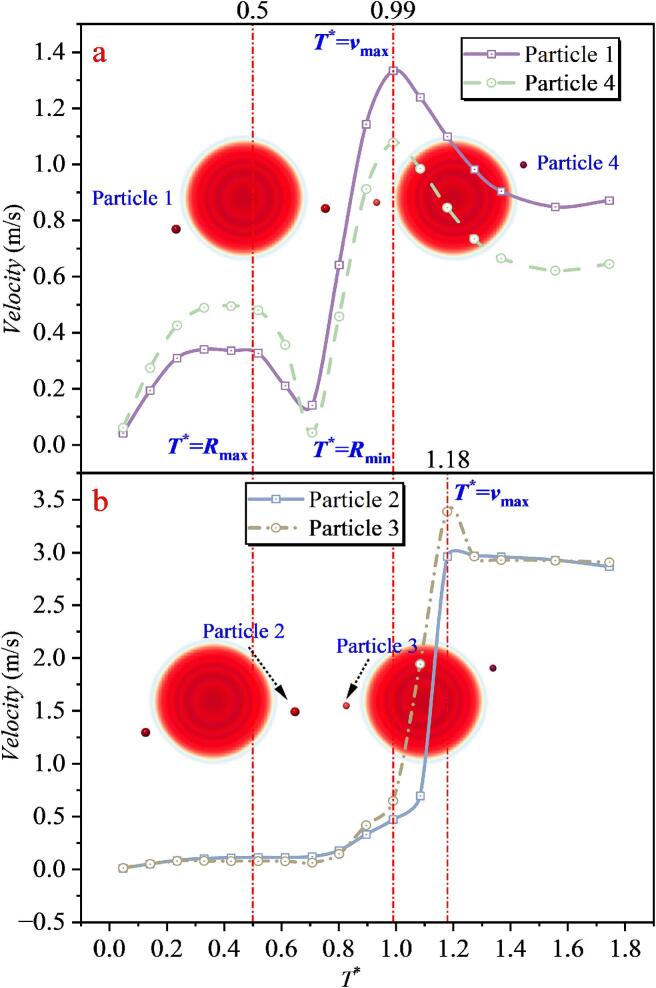
The velocity of particles under the effect of double cavitation bubbles (*λ*_12_ = 1.813) (Case5: *λ*_11p_ = 1.258; *λ*_12p_ = 1.401, λ_22p_ = 2,186; *λ*_13p_ = 2.250, λ_23p_ = 1.308; *λ*_14p_ = 1.355).

When *λ*_12_ increases to 1.813, the microjet-induced impact between the two cavitation bubbles becomes even weaker. As shown in Fig. 21, the maximum velocities of the intermediate particles (Particles 2 and 3) are both below 4 m/s. Combined with the bubble-shape evolution, it is observed that at T* = 0.50, the double cavitation bubble radius reaches its maximum. After this instant, the velocities of Particles 1 and 4 decrease and then increase again, whereas the velocity variations of Particles 2 and 3 remain minor. At T* = 0.99, the double cavitation bubble radius reaches its minimum, and Particles 1 and 4 attain their maximum velocities around this instant. Meanwhile, Particles 3 and 4 reach their peak velocities near T* = 1.18. Notably, clear differences exist in the timing of peak velocities among the particles. This discrepancy is consistently observed in double cavitation bubble systems: different inter-bubble spacings lead to different acceleration effects, and thus the time at which particles reach their maximum velocity also varies accordingly.

**Fig. 22:**
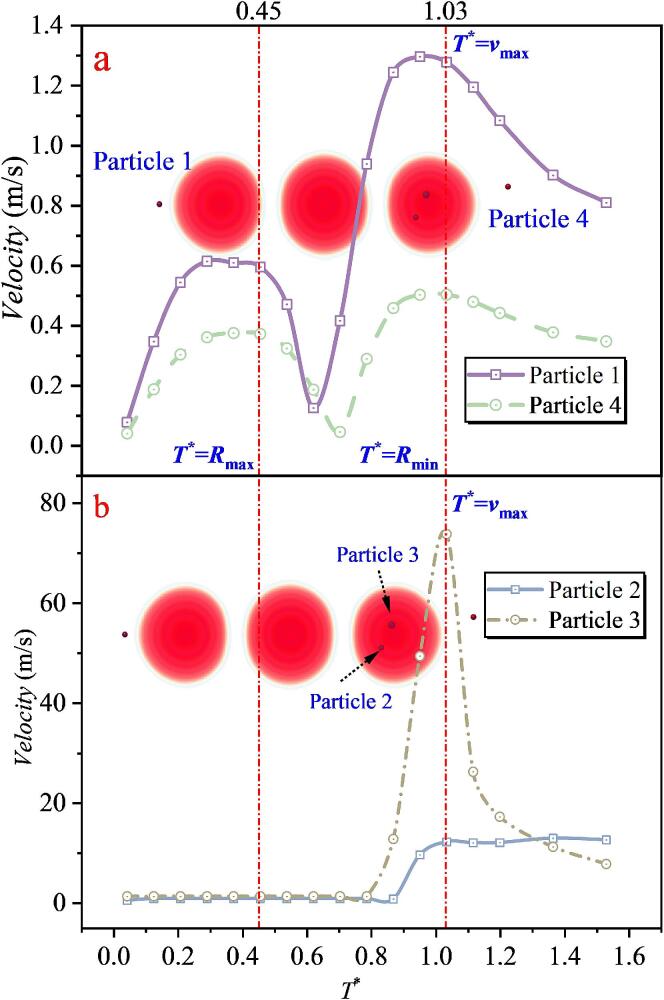
The velocity of particles under the effect of double cavitation bubbles (*λ*_12_ = 1.018, *λ*_23_ = 1.018) (Case6: *λ*_11p_ = 1.263; *λ*_22p_ = 1.851, λ_32p_ = 0.457; *λ*_23p_ = 1.953, λ_33p_ = 0.234; *λ*_34p_ = 1.635).

The horizontally arranged three cavitation bubble configuration represents a special distribution mode of the three-bubble system. Case 6 in Fig. 22 corresponds to the condition where *λ*_12_ and *λ*_23_ are equal. The results show that Particle 1 and Particle 4 in Fig. 22(a) still exhibit velocity characteristics similar to those in the single cavitation bubble case. In contrast, Particles 2 and 3, located between cavitation bubbles 2 and 3, undergo pronounced acceleration. In particular, the maximum velocity of Particle 3 reaches 73.803 m/s, which is far higher than the maximum particle velocity observed in the double cavitation bubble cases, while Particle 2 also reaches 12.983 m/s. By correlating these results with the three-bubble shape evolution, it is found that at T* = 0.45, the three cavitation bubble radius reaches its maximum. After this instant, the velocities of Particles 1 and 4 decrease and then increase again, whereas the velocity variations of Particles 2 and 3 are not pronounced. At T* = 1.03, the three cavitation bubble radius reaches its minimum, and Particles 1–4 attain their maximum velocities around this instant. The peak-velocity times of the four particles are nearly identical, showing almost no time delay.

**Fig. 23:**
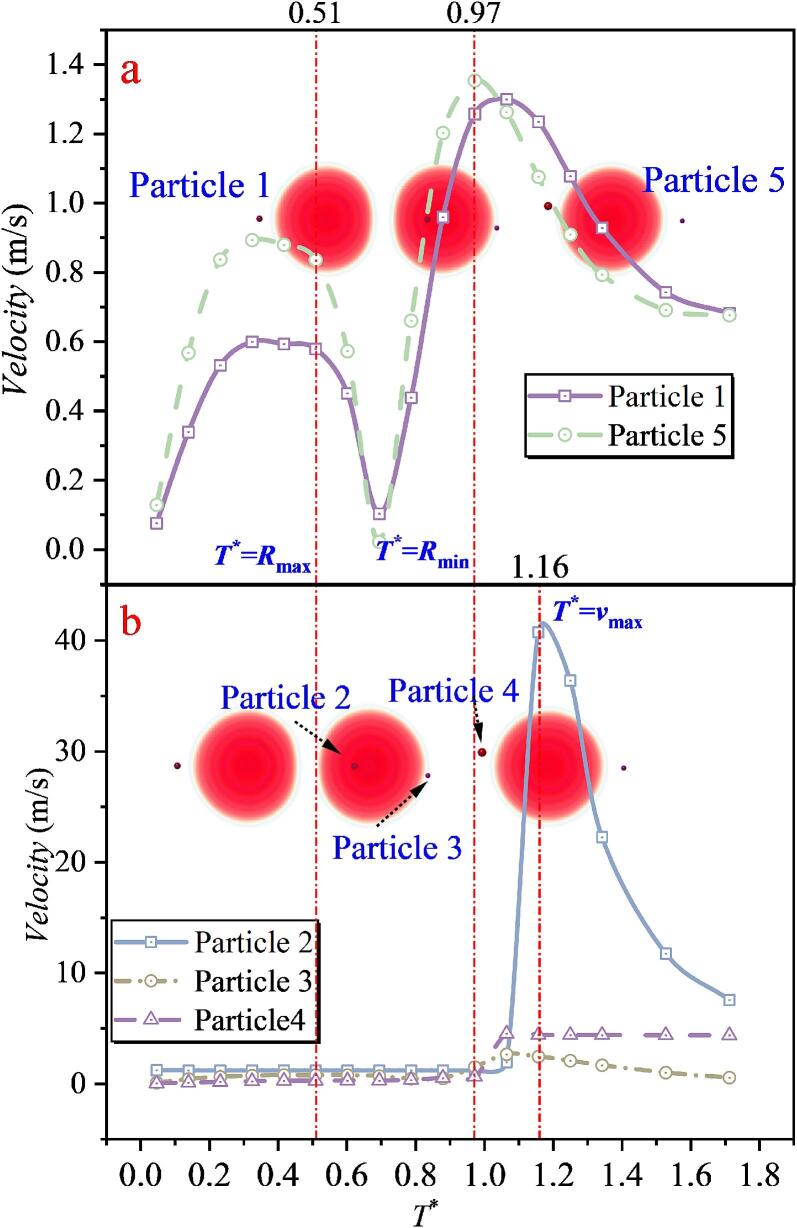
The velocity of particles under the effect of double cavitation bubbles (*λ*_12_ = 1.077, *λ*_23_ = 1.615) (Case7: *λ*_11p_ = 1.295; *λ*_12p_ = 1.898, λ_22p_ = 0.209; *λ*_23p_ = 1.113, λ_33p_ = 2.168; *λ*_24p_ = 1.996, λ_34p_ = 1.177;*λ*_35p_ = 1.297).

When the relative magnitude of *λ*_12_ and *λ*_23_ is then modified, as shown in Fig. 23(a), the velocity characteristics of Particles 1 and 5 remain unchanged. Although the velocities of particles in the intermediate region increase to some extent, pronounced differences emerge. Particle 2, located between cavitation bubbles 1 and 2, is strongly accelerated by the microjet impact and reaches a maximum velocity of 40.751 m/s. By contrast, Particles 3 and 4, located between cavitation bubbles 2 and 3, exhibit maximum velocities below 5 m/s. This clear discrepancy indicates that a smaller spacing between cavitation bubbles 1 and 2 enhances the jet impact and thus accelerates particles more effectively, whereas increasing the spacing between cavitation bubbles 2 and 3 weakens the face-to-face microjet impact, making it difficult to produce substantial acceleration. Under the action of the three cavitation bubble system, due to the unequal spacings, the times at which Particles 2, 3, and 4 reach their maximum velocities differ significantly from those of Particles 1 and 5. This is a direct consequence of the asymmetric three-bubble arrangement.

**Fig. 24:**
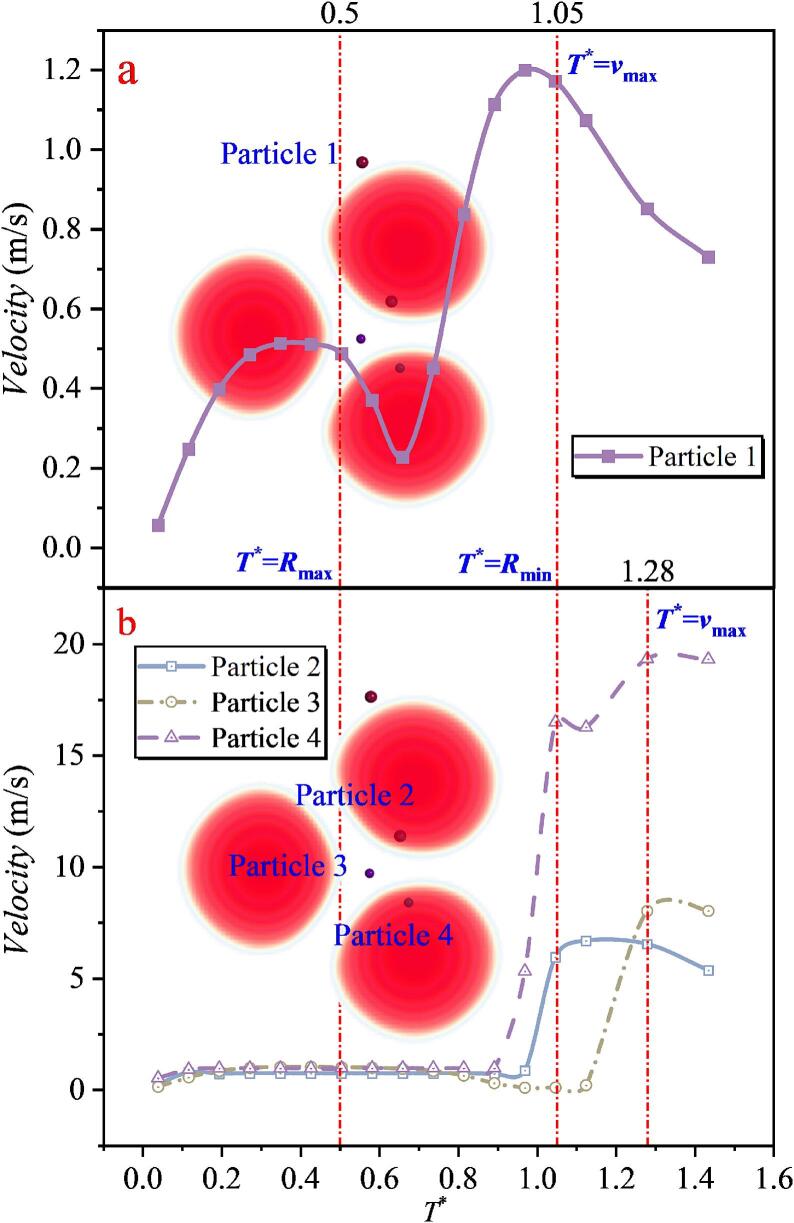
The velocity of particles under the effect of double cavitation bubbles (*λ*_12_ = 1.015, *λ*_23_ = 1.015, *λ*_13_ = 1.015) (Case8: *λ*_11p_ = 1.288; *λ*_12p_ = 0.679, λ_22p_ = 1.420, λ_32p_ = 1.649; *λ*_13p_ = 1.526, λ_23p_ = 1.460, λ_33p_ = 1.572; *λ*_14p_ = 1.470, λ_24p_ = 0.575, λ_34p_ = 1.751).

For the three cavitation bubble system in a triangular configuration, as shown in Fig. 24, when the spacing between any two cavitation bubbles is the same, the maximum velocity of Particle 1 is 1.199 m/s, indicating that it is not subjected to microjet impact. The particles in the intermediate region (Particles 2 and 4) are mainly influenced by cavitation bubbles 1 and 3. In particular, Particle 4 reaches a maximum velocity of 19.339 m/s, but this value is still much lower than the particle velocities observed in the horizontally arranged three cavitation bubble cases. Particle 3 is located almost at the common geometric center of the three cavitation bubbles and reaches a maximum velocity of 8.025 m/s. This suggests that a stronger superposed effect of radiation pressure from multiple cavitation bubbles is, instead, unfavorable for impact-driven particle acceleration; the particle at such a central location exhibits a “locked” behavior. For Particles 1, 2, and 4, the maximum velocities occur at T* = 1.04, when the cavitation bubble radius reaches its minimum. In contrast, due to its special location, Particle 3 reaches its maximum velocity later, at T* = 1.28.

**Fig. 25:**
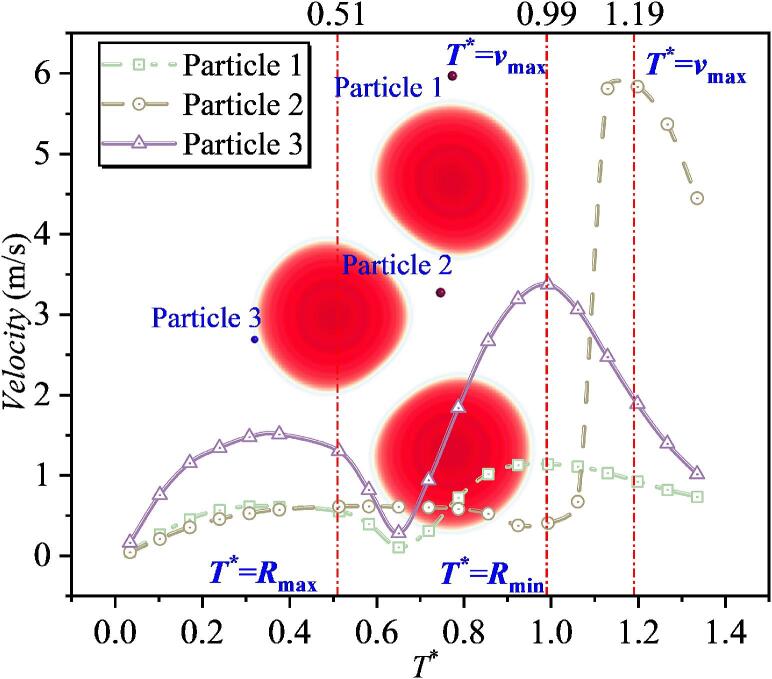
The velocity of particles under the effect of double cavitation bubbles (*λ*_12_ = 1.124, *λ*_23_ = 1.124, *λ*_13_ = 1.621) (Case9: *λ*_11p_ = 1.338; *λ*_12p_ = 1.332, λ_22p_ = 1.308, λ_32p_ = 1.929; *λ*_23p_ = 1.005).

Based on Case 8, the distance between cavitation bubbles 1 and 3 is further increased, as shown in Fig. 25. Particles 1 and 3 exhibit velocity-evolution trends similar to those in the single cavitation bubble case. The particle in the intermediate region (Particle 2) shows a noticeable increase in velocity, reaching a maximum value of 5.835 m/s. However, this value is lower than the peak velocities observed for particles under other three cavitation bubble arrangements. For this condition, it is still observed that the time at which the intermediate particle (Particle 2) reaches its maximum velocity is later than that of the other particles. This timing difference between the central/intermediate particle and particles at other positions is a distinctive feature of the three cavitation bubble configuration.

#### 3.4.3 Impulse statistics of peak events and an impulse-weighted threshold for the affected-range characterization

Discussion of only a few representative particles is not sufficient; an analysis of the overall dynamic characteristics of particle groups is required. In this study, the particle groups contain **N_p_ = 6000** particles, and particle velocities and positions are available at multiple time instants. Here, the particle impulse is defined using a “peak event and the nearest record immediately before the peak” approach. For the *p*_th_ particle, the peak-event time of the y-direction velocity (which corresponds to the bubble-center direction in the double cavitation bubble and selected three cavitation bubble cases) is defined as:

(37) $$t_{p}^{*}=\arg\underset{t}{\max}\left|v_{y,p}\left(t\right)\right|$$

Definition of the peak-event impulse (y-direction):

(38) $$I_{y,p}=m_{p}\left[v_{y,p}\left(t_{p}^{*}\right)-v_{y,p}\left(t_{p}^{-}\right)\right]$$

where $t_{p}^{-}$ is the most recent recorded instant of this particle prior to the peak event, and $m_{p}$ is the particle mass. The y-direction impulse intensity of the particle groups is defined as:

(39) $$I_{y,sum}=\sum_{p=1}^{N_{p}}\left|I_{y,p}\right|$$

To reduce the influence of a single extreme particle on the conclusions, the high-speed set H is defined as the top 5% of $|v_{y}{|}_{max}$(300/6000). The tail-dominance index is defined as:

(40) $$\phi_{I_{p}}=\frac{\sum_{p\in H}\left|I_{y,p}\right|}{\sum_{p=1}^{N_{p}}\left|I_{y,p}\right|}$$

When $\Phi_{I_{y}}$ approaches 1, the group impulse is mainly contributed by a small number of high-speed particles; when $\Phi_{I_{y}}$ decreases, the impulse contribution extends to a broader spectrum of particles. In this study, the initial bubble center is used to define the reference distance at the peak-event instant:

(41) $$\lambda_{\mathrm{ip}}^{\left(0\right)}=\frac{\left\|x_{p}\left(t_{p}^{*}\right)-x_{b0,i}\right\|}{R_{\max}}$$

For multi-bubble systems, the nearest reference distance is adopted:

(42) $$\lambda_{min,p}^{\left(0\right)}=\underset{i}{\min}\lambda_{\mathrm{ip}}^{\left(0\right)}$$

To quantify the affected range of the dominant high-impulse contribution, the cumulative impulse-contribution function over the high-speed set is defined as:

(43) $$F_{I_{y}}\left(\lambda \right)=\frac{\sum_{p\in H,\lambda_{\min,p}^{\left(0\right)}\leq \lambda }\left|I_{y,p}\right|}{\sum_{p\in H}\left|I_{y,p}\right|}$$

The impulse-weighted affected-range threshold is defined as:

(44) $$\lambda_{cr}^{\left(0\right)}\left(0.90\right)=\min\left\{\lambda :F_{I_{y}}\left(\lambda \right)\geq 0.90\right\},r_{cr}=\lambda_{cr}^{\left(0\right)}R_{max}$$

$\lambda_{cr}^{\left(0\right)}$(0.90) does not correspond to the distance of any specific particle; rather, it defines the dominant-contribution range region of the high-speed impulse. Let $r_{\mathrm{cr}}=\lambda_{\mathrm{cr}}^{\left(0\right)}R_{max}$; then, in the three-dimensional free field, this region can be written as:

(51) $$\Omega_{cr}=\left\{x:\underset{i}{\min}\|x-x_{b0,i}\|\leq r_{cr}\right\}$$

A spherical region centered at the initial bubble center of each cavitation bubble with a critical radius *r*_cr_ (for multi-bubble cases, this is defined as the union of multiple such spherical regions). Within this region, the accumulated contribution of high-velocity particles accounts for at least 90% of the y-direction impulse. Therefore, this threshold provides a characteristic spatial scale for the primary source region contributing to the impulse.

Table 4 summarizes the key metrics for Cases 1–9, including the coupling intensity $I_{y,sum}$, the tail-dominance index $\Phi_{I_{y}}$, the dominant-contribution range $\lambda_{cr}^{\left(0\right)}$(0.90), and the concentration index $N_{90}$(the number of high-speed particles required to reach 90% of the cumulative high-impulse contribution).

**Table 4:** Key metrics for all cases.

		*v*_y,95_(m/s)	$I_{y,sum}$(N·s)	$\phi_{I_{y}}$	$\lambda_{cr}^{\left(0\right)}\left(0.90\right)$	$r_{cr}$(mm)	$N_{90}$
Single	Case1	0.028284	1.1923e−5	0.9875	1.3291	9.97	31

Double Cavitation Bubble	Case2	0.074610	2.3326e−5	0.9861	0.6838	5.13	10
	Case3	0.081209	2.2737e−5	0.9478	1.2127	9.09	59
	Case4	0.082033	2.4526e−5	0.9335	1.3783	10.34	84
	Case5	0.089210	3.4352e−5	0.9420	1.4498	10.87	96

Three Cavitation Bubble	Case6	0.119961	3.4160e−5	0.9820	0.8378	6.28	22
	Case7	0.127490	2.6543e−5	0.9695	1.1254	8.44	49
	Case8	0.149010	1.4279e−5	0.9337	1.6823	12.62	142
	Case9	0.493600	1.2472e−4	0.3057	1.7734	13.30	209

For the single cavitation bubble Case1, $I_{y,sum}=1.1923\times 10^{-5}$ N·s and $\lambda_{\mathrm{cr}}^{\left(0\right)}\left(0.90\right)=1.3291$($r_{\mathrm{cr}}=9.97$ mm), with $\Phi_{I_{y}}=0.9875$, indicating that the y-direction group impulse is highly dominated by the high-speed tail, which provides a baseline scale for subsequent multi-bubble comparisons.

For the same-y double cavitation bubble cases, the y-direction momentum-exchange intensity is significantly enhanced relative to the single-bubble baseline ($I_{y,sum}=2.27\times 10^{-5}$–$3.44\times 10^{-5}$ N·s). As the inter-bubble spacing Δy increases from 10 to 25 mm, the dominant-contribution threshold $\lambda_{\mathrm{cr}}^{\left(0\right)}$ increases from 0.6838 to 1.449 (corresponding to $r_{\mathrm{cr}}$ increasing from 5.13 to 10.87 mm), while $N_{90}$ increases from 10 to 96. These results indicate that increasing the double-bubble spacing markedly expands the spatial range of the dominant high-impulse contribution and shifts the contribution toward a larger number of high-speed particles, i.e., increased dispersion.

The collinear three cavitation bubble configurations maintain a high level of tail dominance (Case6: $\Phi_{I_{y}}=0.9820$; Case7: $\Phi_{I_{y}}=0.9695$). Compared with Case7 ($\lambda_{\mathrm{cr}}^{\left(0\right)}=1.1254$, $N_{90}=49$), Case6 ($\lambda_{\mathrm{cr}}^{\left(0\right)}=0.8378$, $N_{90}=22$) exhibits a smaller dominant-contribution range and a higher concentration, attributable to its more symmetric distribution. In contrast, the non-collinear three cavitation bubble configurations show a pronounced increase in both dominant-contribution range and dispersion (Case8: $\lambda_{\mathrm{cr}}^{\left(0\right)}=1.6823$, $N_{90}=142$; Case9: $\lambda_{\mathrm{cr}}^{\left(0\right)}=1.7734$, $N_{90}=209$), indicating that the main contribution range expands outward and is shared by more high-speed particles. Notably, $\Phi_{I_{y}}$ for Case9 (0.3057) is substantially lower than that in other cases, suggesting that the y-direction total impulse is no longer dominated by the top 5% high-speed particles but instead follows a broader-spectrum, collective-contribution mode, implying a potential change in the contribution mechanism under this geometric configuration.

## 4 Conclusion

This study investigates cavitation bubble–particle groups coupling dynamics in a free field, a representative unsteady multiphase problem. Three systems—single cavitation bubble, double cavitation bubble, and three cavitation bubble—are examined by combining experimental observations, theoretical analysis, and three-dimensional numerical simulations to compare particle-group velocity responses. To capture the overall response of particle groups and reduce the bias introduced by isolated extreme particles, a “peak-event impulse statistics” approach is introduced to quantify group-level contributions, together with multiple metrics to enable a unified comparison across bubble numbers and configurations. Based on these multi-scale results, the relative roles of radiation pressure and microjet impact are summarized for different spatial regions and arrangements, providing reproducible evidence for assessing coupling intensity and the affected range in multi-cavitation-bubble–particle-groups systems. The main conclusions are as follows:

The maximum particle velocity decays markedly with increasing dimensionless distance $\lambda_{\mathrm{ip}}$. Two velocity peaks occur during the expansion and collapse stages, respectively, and their relative magnitudes are governed by $\lambda_{\mathrm{ip}}$(the two peaks become comparable when $\lambda_{\mathrm{ip}}\approx 1.8$). The velocity direction points outward from the bubble center during expansion and reverses toward the center during collapse, exhibiting a typical radially oscillatory response. At the group level, the y-direction impulse contribution shows a pronounced “high-speed tail dominance,” serving as a baseline reference for subsequent comparisons with multi-bubble cases.

Particle velocity evolution in the inter-bubble superposition region differs significantly from that in the two outer-side regions. Numerical simulations indicate that, when particles are located near the centerline connecting the two bubble centers, the collapse-induced microjet can trigger peak acceleration events, with peak velocities reaching the order of tens of m/s (e.g., at $\lambda_{12}=0.651$, a small fraction of particles close to the jet core can approach ∼ 60 m/s), which is substantially higher than the velocity range dominated by radiation pressure. Meanwhile, particle velocity directions in the superposition region can become approximately perpendicular to the centerline, whereas the velocity magnitudes and directions in the outer-side regions remain broadly consistent with the single cavitation bubble case. At the group level, the double cavitation bubble configuration markedly enhances the y-direction momentum-exchange intensity relative to the single-bubble case; as the inter-bubble spacing increases, the dominant high-impulse contribution range expands outward, and the contribution pattern transitions from being governed by fewer particles toward a more distributed, collective-contribution mode.

Representative horizontally aligned and triangular configurations show that the relative magnitudes of inter-bubble spacing parameters and the geometric symmetry significantly influence the evolution of particle velocity magnitudes and directions in inter-bubble regions. Symmetric configurations are more likely to exhibit an integral effect, and a small number of representative particles in the intermediate region can be noticeably accelerated by jet impact; when symmetry is broken by changing one inter-bubble spacing, this integral effect weakens and the peak velocities of intermediate particles decrease significantly. At the group level, collinear configurations generally remain strongly tail-dominated, whereas non-collinear configurations exhibit further expansion of the dominant-contribution range together with a pronounced increase in dispersion, and may display (under specific geometries) a transition from “tail dominance” to a broader-spectrum, collective-contribution mode.

In a free field, radiation pressure governs fundamental trends such as the distance-dependent decay of velocity, whereas the microjet impact formed during the collapse stage of multi-cavitation-bubble systems is the key factor responsible for peak-velocity jumps in superposition regions and for the pronounced enhancement of group impulse. The number of cavitation bubbles and their arrangement modulate particle-group responses and contribution patterns by altering the location and intensity of jet formation as well as the momentum-transfer direction within the superposition region.

Numerical model does not account for phase-change mass transfer or the translational degree of freedom of the cavitation bubble; therefore, quantitative predictions of the peak intensity during the collapse stage and the location of microjet action in the superposition region may exhibit deviations. In addition, particles are simplified as regular spheres, without considering irregular shapes or rotational effects. Future work may incorporate phase-change and translation models and further include non-sphericity and rotational dynamics to evaluate how these factors influence the group-level metrics and their applicability bounds.

## CRediT authorship contribution statement

**Shiqi Yang:** Writing – original draft, Software, Methodology, Investigation, Data curation. **Wei Han:** Writing – review & editing, Supervision, Conceptualization. **Rennian Li:** Validation, Resources. **Xiaobo Shen:** Data curation.

## Declaration of competing interest

The authors declare that they have no known competing financial interests or personal relationships that could have appeared to influence the work reported in this paper.

## Glossary

*a*_p_: The particle's acceleration (m**/**s^2^)
*C*_D_: The drag coefficient
*C*_M_: The additional mass coefficient
*F*_add_: The additional mass force (N)
*F*_b_: The gravitational force and the buoyant force combined force (N)
*F*_drag_: The fluid drag force (N)
*F*_inertia_: The flow inertia force (N)
*L*_ijp_: The actual size between the cavitation bubble i and the particle j (mm)
*L*_mn_: The actual size between cavitation bubble m and cavitation bubble n (mm)
*Q*(t): The rate of volume change
*R*_b_(t): The variation of cavitation bubble i radius over time
$\dot{R_{b}}$(t): The first-order derivative of the cavitation bubble radius with respect to time
*R*_imax_: The maximum radius of the cavitation bubble i (mm)
*R*_(m)max_: The maximum radius of the cavitation bubble m (mm)
*R*_(n)max_: The maximum radius of the cavitation bubble n (mm)
*T**: The dimensionless time
***U***: The convergence velocity of the flow field
*U*_r_: The component velocity in the r-direction of the flow field
*U*_z_: The component velocity in the z-direction of the flow field
*v*_p_: The speed of the particles (m/s)
*ρ*_l_: The The density of the fluid
*ρ*_p_: The density of the particle
*λ*_ijp_: The dimensionless distance between cavitation bubble i and particle j
*λ*_mn_: The dimensionless distance between cavitation bubble m and cavitation bubble n
